# Structure–function relationship in FGF signaling: rational design of highly stable FGF2 variants and their effect on cell growth, metabolism, and survival

**DOI:** 10.1186/s13036-026-00723-z

**Published:** 2026-07-01

**Authors:** Karolina Baran, Adolf Koudelka, Katarzyna Kotowska, Aleksandra A. Czyrek, Deborah Beckerova, Martyna Biadun, Vlad-Constantin Ursachi, Radoslaw Karelus, Szymon Sidor, Pooja Dudeja, Daniel Krowarsch, Vladimir Rotrekl, Pavel Krejci, Malgorzata Zakrzewska

**Affiliations:** 1 Department of Protein Engineering, Faculty of Biotechnology, University of Wroclaw, Wroclaw, 50-383 Poland; 2 Department of Biology, Faculty of Medicine, Masaryk University, Brno, 62500 Czech Republic; 3 International Clinical Research Center, St. Anne’s University Hospital, Brno, 65691 Czech Republic; 4 Institute of Animal Physiology and Genetics of the CAS, Brno, 60200 Czech Republic

**Keywords:** Fibroblast growth factor 2, Protein stability, Cell signaling, Stem cells

## Abstract

**Background:**

Fibroblast growth factor 2 (FGF2) is a canonical member of the fibroblast growth factor family with essential roles in embryonic development, tissue regeneration, and stem cell biology, where it supports pluripotency and high proliferative capacity. Consequently, FGF2 is widely used in experimental models of regeneration and stem cell maintenance; however, its rapid thermal degradation at physiological temperatures limits its practical utility and therapeutic potential. Despite the availability of stabilized variants, developing FGF2 analogs that combine exceptional thermodynamic resilience with favorable pharmacokinetic properties remains a critical challenge for stem cell research and regenerative medicine.

**Results:**

Using a combination of consensus sequence analysis and structure-guided protein engineering, we generated novel FGF2 variants with unprecedented thermodynamic stability and optimized extracellular matrix interactions. To enhance bioavailability, we introduced mutations that reduced affinity for heparan sulfate proteoglycans, thereby limiting sequestration within the extracellular matrix. The most stable variant exhibited a denaturation temperature increase of more than 27 °C relative to wild-type FGF2 while fully retaining mitogenic and migratory activity after 5 days of incubation at 70 °C. This exceptional thermal stability was accompanied by markedly increased resistance to proteolytic degradation. In functional cellular models, including adipocyte differentiation and stem cell culture, an optimized long-acting variants demonstrated superior metabolic efficacy and sustained signaling, enabling a reduction in dosing frequency from daily administration to once every three days.

**Conclusions:**

We report the development of highly stable, highly bioactive FGF2 variants that retain full receptor specificity and binding affinity even under extreme conditions. By overcoming the intrinsic instability of the wild-type protein, these engineered FGF2s may enable future stem cell expansion and regenerative therapy applications.

**Supplementary Information:**

The online version contains supplementary material available at https://doi.org/10.1186/s13036-026-00723-z.

## Introduction

FGF2, a member of the mammalian fibroblast growth factor (FGF) family, plays a central role in cellular signaling and regenerative processes. It belongs to the canonical subgroup of 22 FGF family members, which signal through four specific tyrosine kinase receptors (FGFR1–4) in a heparin/heparan sulfate (HS)-dependent manner [1, 2]. FGFR1–3 exhibit additional splice variants (“b” and “c” isoforms), adding further signaling complexity [3]. Alternative translation initiation of FGF2 mRNA produces multiple isoforms with distinct subcellular localization and biological functions [4]. The secreted 18 kDa isoform represents the core therapeutic variant, mediating autocrine and paracrine signaling essential for regenerative medicine [5]. High-molecular-weight (HMW) FGF2 variants (22–34 kDa) localize mainly to the nucleus and interact with nuclear proteins such as Api5, ribosomal protein L6/TAXREB107, and SMN/Sfa66 to regulate apoptosis, ribosome biogenesis, and splicing [6–10]. This isoform diversity enables fine-tuning of diverse cellular responses and highlights the unique therapeutic potential of FGF2.

FGF signaling is ubiquitous and essential throughout life [11]. During embryogenesis, FGFs orchestrate morphogenesis and neurogenesis by regulating cell proliferation, migration, and differentiation. Postnatally, FGF2 functions as a key maintenance factor, driving tissue repair, regeneration, and homeostasis following injury [11–13]. Dysregulation of the FGF–FGFR axis is associated with diverse pathologies, including bone disorders such as craniosynostosis and skeletal dysplasias, chronic kidney disease, and cancer [14, 15]. Beyond its physiological roles, FGF2 is a central reagent in stem cell biology. It is the essential mitogen in pluripotent stem cell (PSC) culture media, sustaining self-renewal and maintaining pluripotency in human embryonic stem cells (hESCs), induced pluripotent stem cells (iPSCs), and mouse epiblast stem cells (EpiSCs) [16–18].

FGF2’s potent regenerative, angiogenic, and anti-apoptotic properties have been leveraged in diverse clinical contexts. Topical application of recombinant FGF2 (Trafermin) is approved in regulated markets, including Japan, for the treatment of burns and chronic skin ulcers, such as diabetic foot ulcers [19–22]. In addition, FGF2 has shown promise in regenerating hard tissues, including bone and periodontal structures [23–26], and exhibits neuroprotective effects by stimulating neurogenesis in the adult brain [27, 28].

Despite its therapeutic potential, the utility of native FGF2 is limited by poor biophysical stability [29]. The protein degrades rapidly at physiological temperature (37 °C), resulting in a short half-life that necessitates frequent dosing or complex sustained-release formulations [28]. High affinity of wild-type FGF2 for extracellular matrix proteoglycans can further restrict tissue penetration and bioavailability. Enhancing the physicochemical stability while preserving biological activity remains a major challenge in protein-based therapeutics [30]. Consequently, there is a growing number of approaches aimed at overcoming these problems by improving half-life and pharmacokinetics, as well as by selecting appropriate storage conditions and formulation.

For example, protein engineering has been used to develop a variant of FGF2 with enhanced thermal stability [31]. It has been observed that the improved thermodynamic parameters result in reduced dependence on the presence of heparin, which may be of significant importance for medical applications [32]. Another strategy involves chemical modification, such as PEGylation [33]. PEGylated FGF2 has demonstrated neuroprotective effects and functional improvement in models of Parkinson’s disease, likely due to its prolonged half-life and reduced immunogenicity [34]. While PEGylation enhances pharmacokinetic properties, it may also introduce limitations, including steric hindrance that can compromise biological activity. Another approach involves the use of fusion constructs, such as the chimeric FGF1:FGF2 protein, which is characterized by increased stability and enhanced biological activity [35]. While such constructs offer distinctive advantages, including broader specificity toward FGF receptors, their design and safety evaluation can be more complex. Finally, formulation-based strategies, such as the adsorption of FGF2 onto albumin/heparin complexes [36] or its stabilization against thermal and processing stressors [37], are employed to improve stability and bioavailability in specific administration contexts.

In this study, we employed a rational design strategy to generate next-generation FGF2 variants optimized for biomedical applications. Building on our previous success in stabilizing FGF1 [38] and FGF10 [39], we combined homologous sequence analysis with structural data to design FGF2 variants exhibiting extreme thermal stability and resistance to proteolytic degradation. To improve biodistribution and efficacy, specific mutations were introduced to reduce heparin/heparan sulfate affinity, effectively decoupling protein stability from matrix sequestration. This approach yielded highly potent, long-acting growth factors suitable for regenerative medicine and stem cell applications.

## Materials and methods

### Design of stable FGF2 variants

Stable FGF2 variants were designed using a consensus approach [40] and rational engineering based on structures deposited in Protein Data Bank. Sequences of fibroblast growth factor family proteins were obtained from the Pfam database [41] and ClustalW software was used to align their sequences [42]. Amino acids involved in binding to the receptor and heparin were identified using LigPlot software, based on FGF2 complexes with heparin (1BFB), FGFR1 (1CVS) and heparin and FGFR1 (1FQ9) [43]. To determine the positions of the amino acids that form β-turns and β-strands, PROCHECK software was used [44]. To improve the stability of the protein, amino acids with greater potential for forming secondary structures were introduced, whilst avoiding substitutions that would interfere with interactions with the receptor, heparin or the local hydrogen bond network. The possibility of local steric conflicts was verified using Swiss-PdbViewer and the built-in side chain rotamer database. In addition, two exposed and reactive cysteines (C78 and C96) were replaced with serines to prevent dimer formation. Single mutations giving the greatest increase in thermal stability were used to generate multiple variants. In the next step, selected lysine and arginine residues (K128, R129, K134) were mutated to reduce affinity for heparin.

### Expression and purification of recombinant proteins

FGF2 constructs in the pET-3d vector (GeneUniversal, USA) containing a DNA fragment encoding FGF2_1−155_ or Met-Ala-FGF2_25–155_ were used to express full-length (long) or truncated (short) versions of FGF2 variants, respectively. Expression and purification of FGF2 variants were carried out as follows: *E. coli* BL21(DE3)pLysS cells (Merck, Germany) were transformed with plasmids containing FGF2 variant sequences and cultured at 37 °C to OD_600_ 1.0 in TB medium (BioShop, Canada) supplemented with 1% glucose (BioShop, Canada), 100 µg/mL ampicillin (Polfa Tarchomin, Poland) and 30 µg/mL chloramphenicol (BioShop, Canada). Protein expression was induced by adding IPTG (Iris Biotech, Germany) to a final concentration of 1 mM. After 16 h of overnight culture at 25 °C, the bacteria were harvested. The bacterial pellet was resuspended in lysis buffer (50 mM NaH_2_PO_4_, 0.15 M NaCl, 1 mM DTT, 1 mM EDTA, 0.1 mM PMSF, 0.1% Triton X-100, pH 7.2), sonicated and centrifuged (14500x g, 40 min). The resulting supernatant was further diluted with wash buffer (50 mM NaH_2_PO_4_, 0.7 M NaCl, 1 mM DTT, 1 mM EDTA, 0.1% Triton X-100, pH 7.2) to a final NaCl concentration of 0.5 M. Proteins were purified from the soluble fraction using a HiTrap Heparin HP column (Cytiva, UK) with a linear gradient of 0.5-2 M NaCl. Recombinant proteins were analyzed by SDS-PAGE and mass spectrometry to confirm purity and identity.

### Biophysical characterization of FGF2 variants

Jasco J-1500 spectropolarimeter (Jasco, Japan) was used to determine the proper folding of proteins. Ellipticity was measured in the wavelength range 200–260 nm in 25 mM phosphate buffer (pH 7.3) at protein concentration of 5 µM. Spectra were collected in a cuvette with an optical path length of 2 mm. To determine the stability of the FGF2 mutants, thermal denaturation experiments were carried out analyzing changes in ellipticity at 228 nm while heating the samples at a constant rate of 1 °C/min. The temperature range for each measurement was set to at least ± 30 °C relative to the denaturation temperature of the specific protein variant. The experiments were performed at a protein concentration of 0.5 µM, a slit width of 2 nm and a response time of 8 s. Temperature was monitored directly in the protein solution. In addition, selected measurements were also performed in the presence of 1 µM heparin (Sigma Aldrich, USA). Data were analyzed using PeakFit (Systat, USA) with a two-state denaturation model.

To determine the binding affinity of different FGF2 variants to heparin, HiTrap Heparin HP column and NGC Chromatography System (BioRad, USA) were applied. The recombinant proteins were loaded onto a heparin column at a concentration of 0.2 mg/mL and then eluted in a linear gradient of 0–2 M NaCl, 50 mM Na_2_HPO_4_ (pH 7.2). The NaCl concentration at which the protein was released from the column was determined based on conductivity using ChromLab software.

To verify the susceptibility of stable FGF2 variants to proteolytic degradation, they were subjected to a limited proteolysis assay at 37 °C in a buffer containing: 100 mM Tris-HCl, 20 mM CaCl_2_, pH 8.3, in the presence of trypsin with a trypsin: protein molar ratio of 1:50. The reaction was stopped with Laemmli sample buffer at 95 °C for 10 min. Next, the samples were analyzed by SDS-PAGE.

Biolayer interferometry (BLI) was applied to measure the interaction of FGF2 variants with FGFR1c. The fully glycosylated extracellular domain of FGFR1c fused to the Fc fragment of IgG1 was purified from CHO cells according to the procedure described before [45]. FGFR1c-Fc was immobilized on a protein A biosensor and incubated with FGF2 variants diluted in PBS at concentrations ranging from 4 to 512 nM, in the presence or absence of 1 µM heparin. Association and dissociation were monitored for 180 s each using Octet K2 (Sartorius AG, UK). The resulting data were analyzed using ForteBio Data Analysis 11.0 software (Sartorius AG, UK). Equilibrium dissociation constants (K_D_) were determined using saturation binding curves.

### Cell culture and biological activity assays

NIH 3T3 cells (ATCC, USA, CRL-1658) were propagated in DMEM medium (BioWest, USA) supplemented with 10% FBS (Gibco, Thermo Fisher Scientific, USA). 3T3-L1 cells (ATCC, CL-173) were differentiated by a 3-day induction with DMEM buffered with NaHCO_3_ (PAN-Biotech GmbH, Germany) and supplemented with 10% FBS, 0.5 mM isobutylmethylxanthine (Sigma Aldrich, USA), 1 µg/mL insulin (R&D Systems, USA) and 1 µM dexamethasone (Sigma Aldrich, USA), and then matured in DMEM supplemented with 10% FBS and 1 µg/mL of insulin until day 12. Human osteosarcoma U2OS cells stably transfected with wild-type FGFR1c (U2OS_R1) were maintained in DMEM medium with 10% FBS, as described previously [46].

Rat chondrosarcoma (RCS) cells were cultivated in DMEM medium supplemented with 10% FBS. RCS cells expressing single isoforms of FGFRs: FGFR1c, FGFR2c, FGFR3c or FGFR4, were generated from wild-type RCS cells by CRISPR/Cas9 inactivation of unwanted endogenous FGFRs [47]. To obtain lines with a single FGFR “b” isoform (FGFR1b, FGFR2b, or FGFR3b) *Fgfr1-4 null* cells were generated by CRISPR/Cas9 and stably transfected with vectors containing a single V5-tagged full-length human FGFR1-3b. Stable integration was achieved by PiggyBac transposase, and low FGFR expression was ensured by the attenuated CMV promoter [47]. To assess FGF2-induced cell signaling, the western blot method was used. Cells were harvested directly into Laemmli sample buffer and lysates were separated by SDS-PAGE, transferred to PVDF membrane, and signaling proteins were detected using specific primary antibodies and HRP-conjugated secondary antibodies. Protein-specific bands were visualized by chemiluminescence. Western blot signal was quantified using ImageJ software [48]. Table S1 lists all used antibodies. Wild-type FGF1 (used as a control protein) was produced as described before [38] or purchased from R&D Systems (USA).

For growth arrest analysis, RCS cells were seeded onto a 24-well plate in complete medium. Wild-type FGF2 and its stable variants were added at concentrations of 0.2, 0.5, 1, 5, 10 and 20 ng/mL. After 96 h, cells were trypsinized and counted using a Beckman Coulter Cell Counter (Beckman, USA).

To assess mitogenicity, NIH 3T3 cells were seeded into 96-well plates. After overnight starvation, the cells were treated with fresh or challenged (incubated at elevated temperature) FGF2 multiple mutants in the concentration range of 0.1–100 ng/mL in the presence of 10 U/mL of heparin (Sigma Aldrich, USA). After 48 h, the metabolic activity of the cells was assessed using AlamarBlue (Thermo Fisher Scientific, USA). The results were normalized using the maximum response of FGF2 wild-type (100 ng/mL) as 100%.

In the wound scratch assay, NIH 3T3 cells were seeded onto 96-well ImageLock plates and serum-starved for 24 h. Then, cells were scratched with WoundMaker (Essen BioScience, Germany) and incubated in serum-free medium supplemented with 10 ng/mL of wild-type or mutants of FGF2 for 36 h [49]. Wound images were acquired every 2 h using IncuCyte ZOOM System (Essen BioScience, Germany).

In the anti-apoptotic activity assay, NIH 3T3 cells were serum-starved for 24 h and then incubated for 24 h with freshly prepared or challenged (48 h at 70 °C) wild-type or mutants of FGF2 (10 ng/mL) in the presence of 10 U/mL heparin [49]. Cell viability and caspase-3/7 activity were measured using the ApoLive-Glo Multiplex Assay (Promega, USA) according to the manufacturer’s protocol. The ratio of caspase-3/7 activity to cell viability was normalized against untreated cells.

Metabolic activity of FGF2 variants was characterized in 3T3-L1 adipocytes. Differentiated 3T3-L1 adipocytes were seeded onto a 96-well BioCoat™ Poly-D-Lysine plate (Corning, USA) in DMEM without glucose (Thermo Fisher Scientific, USA) and stimulated with 10 ng/mL of fresh or challenged FGF2 proteins in the presence of 10 U/mL of heparin for 18 h [50]. Glucose uptake was then determined using the Glucose Uptake-Glo™ Assay (Promega, USA) according to the manufacturer’s protocol. Results were normalized and expressed as a percentage of the signal generated by fresh wild-type FGF2.

### Stem cells differentiation and quantitative real-time PCR

To evaluate the effect of FGF2 variants on stem cell pluripotency, we utilized the human induced pluripotent stem cell line cl.4 obtained from Dr. Majlinda Lako (Newcastle University, UK) [51]. Cells were cultured long-term in the form of colonies on inactivated mouse embryonic fibroblasts (MEF) and for experiments on Matrigel hESC-qualified Matrix (Corning, USA) coated well plates in conditioned media (CM) as described previously [52, 53]. Briefly, for feeder culture, cells were fed with human embryonic stem cell media (HES; Thermo Fisher Scientific, USA) supplemented with 15% knockout serum replacement (Thermo Fisher Scientific, USA), 1% non-essential amino acids (Thermo Fisher Scientific, USA), 1% L-glutamine (Biosera, France), 0.5% penicillin–streptomycin (Biosera, France), 2-mercaptoethanol (Sigma-Aldrich, USA) and 10 ng/mL FGF2 (PeproTech, Thermo Fisher Scientific, USA). For feeder-free culture, HES media without FGF was conditioned on MEF for 24 h at a time with CM collected and replaced by fresh media for seven days. Collected CM was mixed, supplemented with L-glutamine and filtered. No FGF2 (control) or one of the FGF2 variants was added at 10 ng/mL. Cells were passaged enzymatically using TrypLE Express Enzyme (Thermo Fisher Scientific, USA). Media were changed every day or every three days. To investigate the effect of FGF2 variants on differentiation, cl.4 cells were cultured for three passages in CM medium supplemented with 100 ng/mL of wild-type, 8XC2, or 8XC2H1 FGF2. Fragments of the monolayer were manually scraped from the confluent dish and then allowed to form embryoid bodies in MEF medium. The medium was changed every three days, and samples were collected for RT-qPCR. Total RNA was isolated using the RNA Blue reagent (Top-Bio, Czech Republic) according to the manufacturer’s instructions. The concentration and purity of messenger RNA (mRNA) were determined using NanoDrop (Thermo Fisher Scientific, USA). Two micrograms of total RNA were transcribed into complementary DNA (cDNA) using the Moloney Mouse Leukemia Virus (M-MLV) reverse transcriptase (Invitrogen, Thermo Fisher Scientific, USA) and Oligo(dT) primers (Thermo Fisher Scientific, USA) at 37 °C for 1 h followed by 5 min at 85 °C. Quantitative real-time PCR (qRT-PCR) was performed using the TP SYBR 2x Master Mix (Top-Bio, Czech Republic) in a Light Cycler 480 instrument (Roche, Switzerland). The sequences of primers used are presented in Table S2. The resulting data were normalized to glyceraldehyde 3-phosphate dehydrogenase (GAPDH) mRNA expression and presented as 2^−Δcq^.

### Statistical analysis and adjustment of microphotographs

All experiments were performed in at least triplicate. The number of independent experiments (n), the method of data generation (in the bar and line graphs) and the method of statistical analysis of the data are indicated in each figure legend. Brightness and contrast were set uniformly in the microscopic photographs in each panel.

## Results

### Engineering of thermally stable FGF2 variants

Recombined FGF2 proteins were produced in two versions, differing in the length of the N-terminus of the protein (long FGF2_1−155_ and short FGF2_Met−Ala−25−155_). The differences in stability between these constructs, for both wild-type and mutant proteins, were minimal, usually less than 1 °C (Table S3). However, we observed a substantial increase in protein expression level for the “short” version of FGF2. Thus, most of the FGF2 mutants were constructed based on FGF2_short_. For the final multiple mutant variants, we worked with the “long”, naturally occurring, form of the protein.

**Fig. 1:**
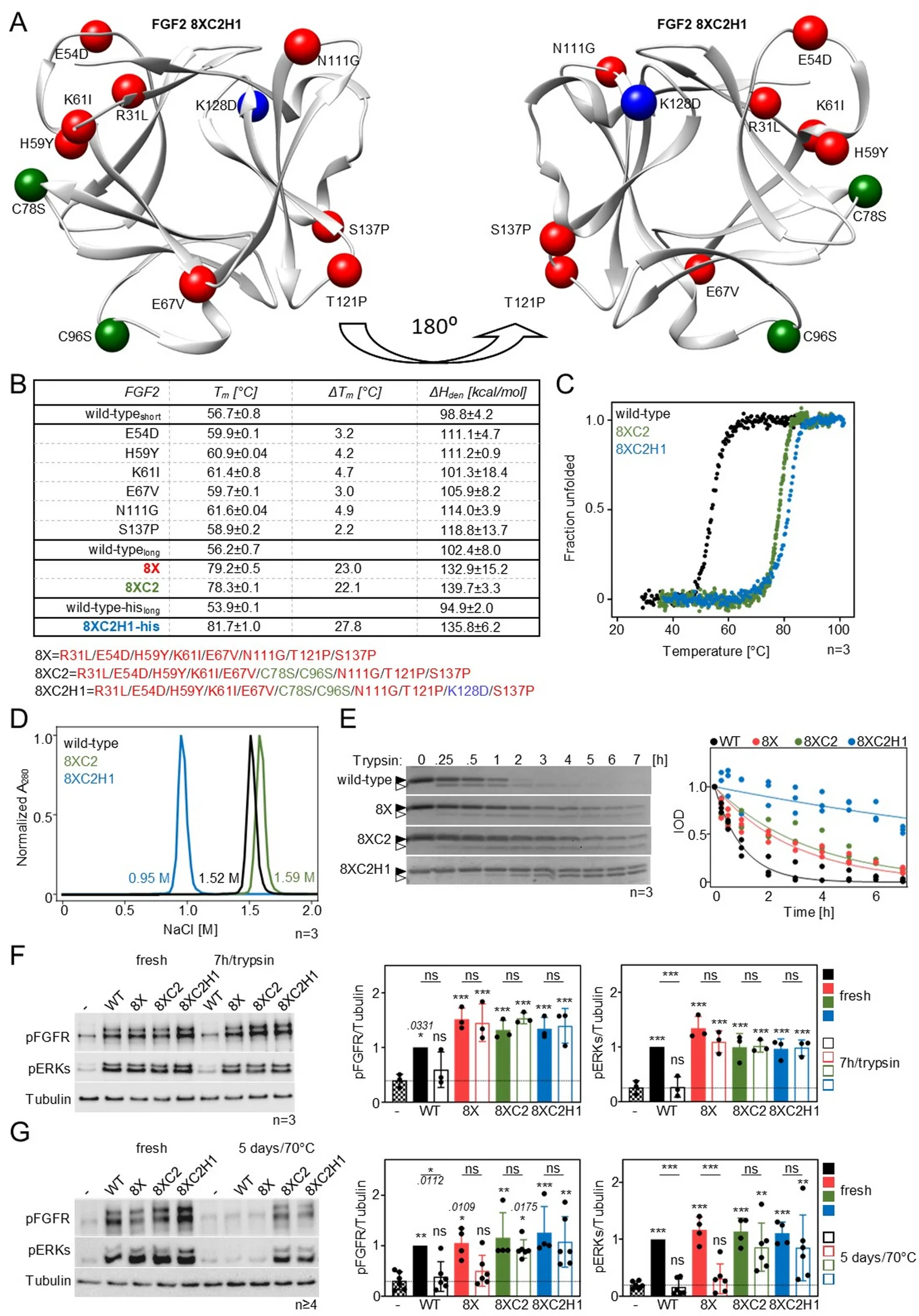
Development of stable FGF2. (**A**) Model of stable FGF2. Stabilizing substitutions are marked in red, cysteine-eliminating substitutions are shown in green, and heparan affinity-reducing mutations are shown in blue. (**B**) Thermal stability of FGF2 variants determined by circular dichroism (CD) spectroscopy; *n* = 3; mean ± SD; *T*_*m*_ melting temperature; *H*_*den*_ denaturation enthalpy. (**C**) Thermal denaturation curves of FGF2 variants. Changes in FGF2 structure determined with CD by monitoring ellipticity at 228 nm with increasing temperature (30–100 °C); *n* = 3. (**D**) Representative elution profile of multiple FGF2 variants from HiTrap Heparin HP column, obtained in a gradient of 0.05–2.05 M of NaCl; *n* = 3. The values on the graph represent the NaCl concentration at which each protein elutes. (**E**) Resistance of FGF2 variants to proteolytic degradation. FGF2 muteins were incubated with trypsin for the indicated times and analyzed by SDS-PAGE. Black arrowhead indicates full-length FGF2; white arrowheads, intermediate products of trypsin proteolysis. Wild-type FGF2 is completely degraded within 2 h, while stabilized variants are detectable after 7 h of incubation. Dots in densitometric analysis represent individual experimental replicates, lines are linear regressions; *n* = 3. (**F**) Activity of FGF2 variants in NIH 3T3 cells after incubation with trypsin. FGF2 variants were pre-incubated with trypsin for 7 h before being added to the cells for 15 min. Phosphorylated (p) ERKs and FGFR were detected by western blot, with the total tubulin serving as a loading control. Densitometric analysis of pERKs and pFGFR signal are shown, values relative to stimulation with fresh wild-type protein (mean ± SD; one-way ANOVA, *p ≤ 0.05, ***p ≤ 0.001; *n* = 3). (**G**) Activity of FGF2 in NIH 3T3 cells after preincubation for 5 days at 70 °C. Pre-incubated FGF2 variants were added to the cells for 15 min. Phosphorylated (p) ERKs and FGFR were detected by western blot, with total tubulin serving as a loading control. Densitometric analysis of pERKs and pFGFR signal are shown, values relative to stimulation with fresh wild-type protein (mean ± SD; one-way ANOVA, *p ≤ 0.05, **p ≤ 0.01, ***p ≤ 0.001; *n* ≥ 4)

To enhance the thermal stability of FGF2, we designed 43 single-point mutants using a rational approach based on 3D structure, consensus strategy and template-based thermostability engineering. Forty of these variants were successfully overexpressed in a bacterial expression system and subsequently purified. We collected circular dichroism (CD) spectra to verify their proper conformation and performed stability analysis by measuring the signal change at 228 nm. Among the variants, 20 exhibited increased stability relative to the wild-type protein,

16 were less stable, and 4 were unfolded and lacked cooperative denaturation profiles (Table S4, S5; Fig. S1A). The most stable single variants were further evaluated for stability in cell-conditioned medium and their ability to activate FGFR-dependent signaling pathways (Fig. S1B, C). Based on the stability data, we combined mutations that conferred substantial stabilization to generate nine multiple FGF2_short_ variants (Table S6). Among these, the variant carrying mutations: R31L/E54D/H59Y/K61I/E67V/N111G/T121P/S137P (designated 8X, Fig. 1A, B) showed the highest denaturation temperature (79.3 °C), representing an increase of 22.6 °C compared to the wild-type protein (Table S6). All nine variants were biologically competent, activating ERK pathway (Fig. S2).

### Design and characterization of FGF2 variants with modified heparin affinity and proteolytic resistance

In the next step, based on the structure of FGF2 in complex with FGFR1 and heparin (1FQ9) [43], we designed eight FGF2_short_ variants (K128N/D, R129G, K134E/V, Q143N, K144P, K145D) with impaired affinity for heparin. Finally, we selected four variants (single and double) for which we verified thermodynamic stability and affinity for heparin (Fig. S3; Table S7). Three substitutions (K128D, K134V, R129G) were further combined with the stable 8X variant (Table S8). A multiple variant containing three mutations disrupting heparin binding, showed a five-fold decrease in heparin affinity. Nevertheless, it exhibited a significant decline in biological activity and was therefore excluded from further analysis (Table S8, Fig. S3).

It was previously shown that substitution of cysteine residues in FGF1 significantly prolongs the half-life of the protein in in vitro assays [54]. Therefore, to further increase the half-life of multiple FGF2 variants, we included cysteine to serine mutations in the most stable constructs. Finally, we designed 8 FGF2_long_ variants containing stabilizing mutations (8X) in combination with cysteine-to-serine mutations and heparin affinity disturbing substitutions (Fig. 1A, C, D; Fig. S4; Table S9).

### Biophysical characterization and in vitro stability of engineered FGF2 variants

Based on thermodynamic analysis, degradation experiments and heparin binding profiles, we selected three variants for further analysis (8X, 8XC2, 8XC2H1). The mutants exhibited remarkable properties compared to the wild-type FGF2. Spectroscopic analysis confirmed the structural stability of the selected variants during incubation at 37 °C and 50 °C. All variants retained the conformation characteristic of the β-trefoil structure for 5 days at 37 °C, whereas the wild-type protein lost its three-dimensional structure within 24 h at 50 °C [55]. In contrast, the stabilized variants retained their intact structure throughout the duration of the experiment. In protein degradation experiments, using trypsin as a model enzyme, we observed intact, stable FGF2 mutants that remained biologically active even after 7 h of proteolysis, while the wild-type was almost completely degraded after 2 h. The half-lives of WT, 8X, 8XC2, and 8XC2H1 in the trypsinolysis assay were 19 min, 55 min, 65 min, and 351 min (nearly 4 h), respectively (Fig. 1E).

We developed an assay to validate the activity of FGF2 mutants exposed to trypsin or high temperature (70 °C) in order to assess their suitability under adverse conditions. The 8XC2 and 8XC2H1 mutants remained structurally intact, possessing the ability to activate the receptor and downstream signaling pathway (ERK1/2 phosphorylation) after incubation with trypsin for 7 h (Fig. 1F) and after five days of incubation at 70 °C (Fig. 1G). Wild-type FGF2 treated in the same manner completely lost its activity (Fig. 1F, G). Furthermore, wild-type FGF2 incubated in media with differentiated 3T3-L1 adipocytes ceased to be biologically active after 48 h, while the stable 8XC2 mutant showed the ability to activate ERKs even after 120 h (Fig S5).

### Interaction of stable FGF2 variants with the FGF receptor 1

**Fig. 2:**
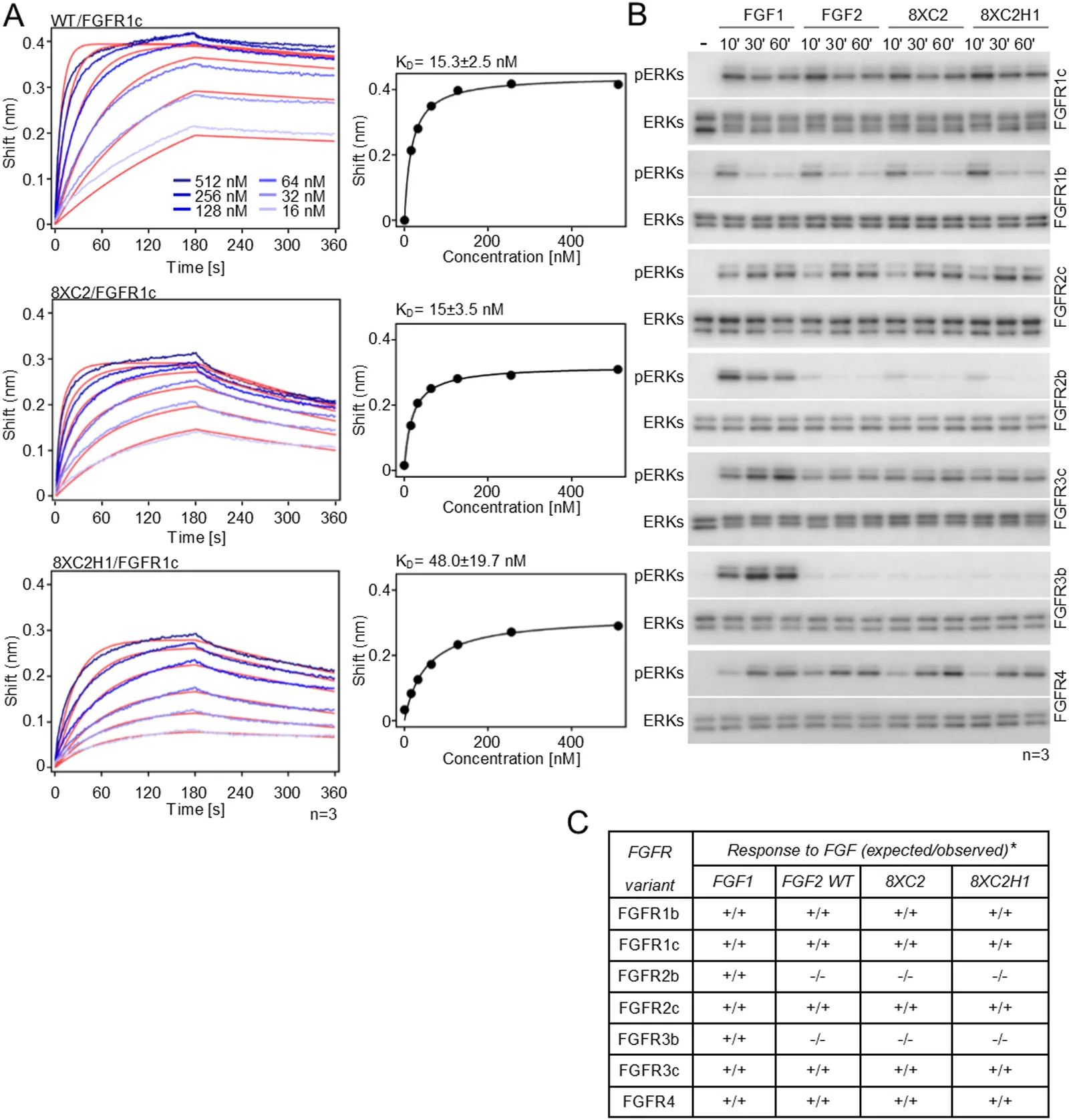
Specificity of stable FGF2 variants. (**A**) BLI analysis of the affinity of FGF2 variants for FGFR1-Fc (IIIc isoform). FGFR1-Fc was immobilized on a ProA sensor and its interactions (association and dissociation) with selected FGF2 mutants in the concentration range 16–512 nM were analyzed. Curves obtained by global fitting are marked in red. Representative results are shown. The equilibrium dissociation constant (K_D_) was calculated for steady state conditions; *n* = 3. (**B**) RCS cells expressing individual FGFR variants were treated with FGF1, wild-type FGF2 and its stable variants at the indicated times (10–60 min), and then analyzed for phosphorylation (p) of ERKs; total ERKs level was used as a loading control. (**C**) Summary of western blot data obtained (**B**) (observed), compared with published data (expected; [56, 89])

FGF2 protein interacts with cells by binding to the following FGFR isoforms: FGFR1c, FGFR3c, FGFR2c, FGFR1b and FGFR4 [56]. To confirm that the introduced amino acid substitutions do not affect the specificity and affinity of FGF2 variants to FGFR1c, we performed biolayer interferometry analysis (BLI, Fig. 2A). The extracellular domain of FGFR1c in fusion with the Fc fragment was immobilized on a ProA sensor, and the association and dissociation of selected FGF2 variants were analyzed. Equilibrium dissociation constants (K_D_) were calculated using saturation binding curves. The data obtained showed that the stable FGF2 variant, 8XC2, binds FGFR1c with a strength similar to that of the wild-type protein, while for 8XC2H1 (with impaired binding to heparin, the stabilizing co-factor of the FGF: FGFR complex) the binding was only slightly attenuated (K_D_=15.3 ± 2.5 nM and K_D_= 48 ± 19.7 nM, for wild-type and 8XC2H1, respectively). The addition of 1 µM heparin had no significant effect on the dissociation constants (Table S12).

In addition, to confirm the FGFR specificity of FGF2 variants in vitro, we generated cell lines with defined FGFR expression. Rat chondrosarcoma (RCS) cells, commonly used to investigate FGF signaling [57–59], express four FGF-receptors (FGFR1–3 “c” and FGFR4). Using CRISPR/Cas9 technology, we removed selected FGFRs, yielding RCS cells endogenously expressing only one receptor (FGFR1c, FGFR2c, FGFR3c or FGFR4). To obtain the FGFR “b” isoform cell lines, we stably transfected FGFR1–4 null RCS cells with full-length human FGFR1-3b vectors [47]. The resulting seven cell lines expressing single FGFR variants, i.e., FGFR1c, FGFR1b, FGFR2c, FGFR2b, FGFR3c, FGFR3b, and FGFR4, were stimulated with stable FGF2 variants, and FGF-dependent activation of ERKs was determined (Fig. 2B, Fig. S10). All FGF2 mutants tested activated the FGFR variants according to expected preferences [56] (Fig. 2C) and kinetics. FGF1, which can activate all FGFR isoforms, was used as a positive control of the obtained cell models.

### Thermal stability mimics the protective effect of heparin

**Fig. 3:**
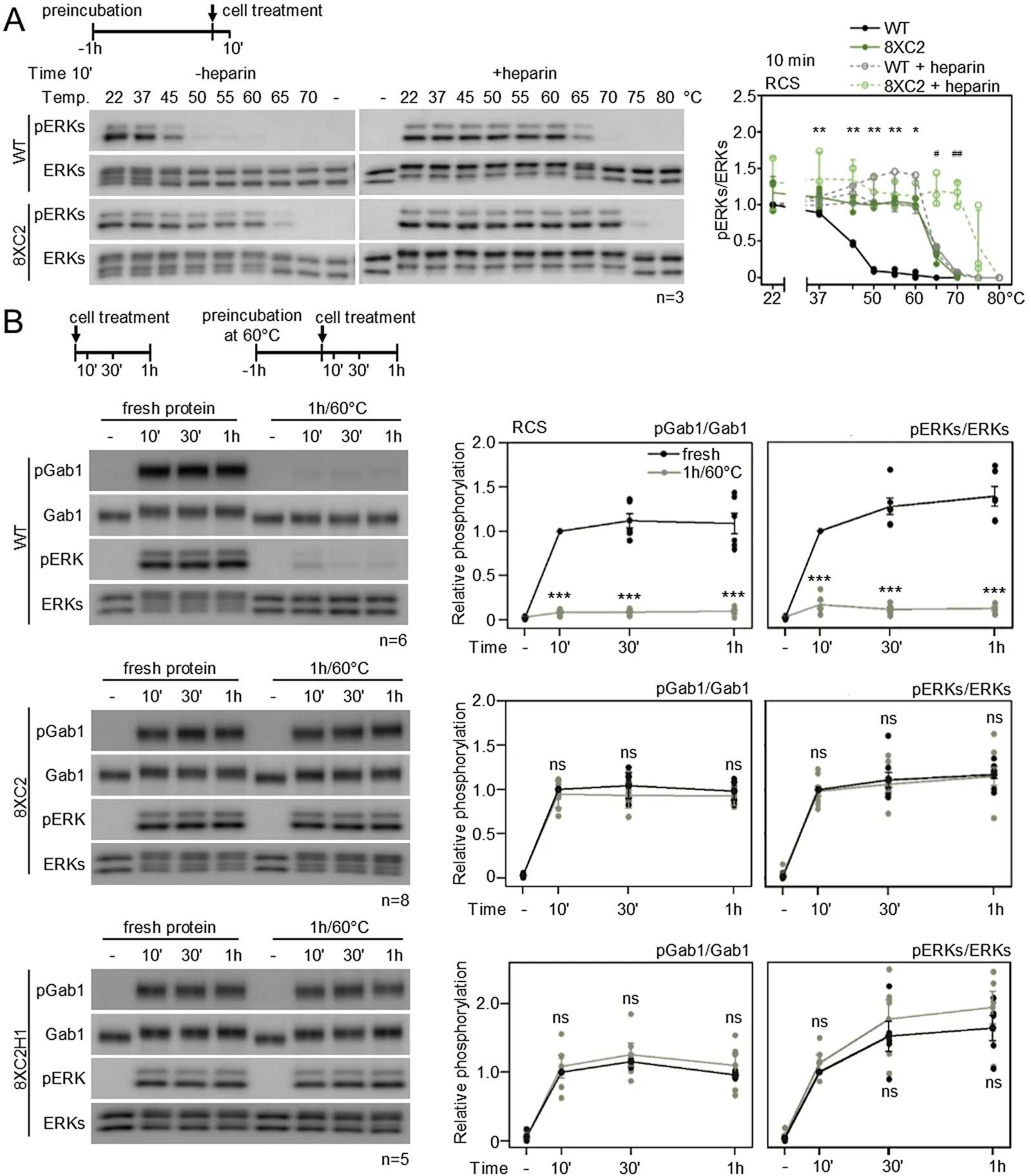
The enhanced thermal stability of the modified FGF2 allows it to maintain its signaling activity in RCS cells. (**A**) RCS cells were treated with 20 ng/mL of pre-incubated (in the presence or absence of 1 µg/mL of heparin) FGF2 variants for 10 min. Phosphorylated (p) ERKs were detected by western blot; total ERKs served as a loading control. Densitometric analyses of the phosphorylated (p) protein signal are shown, with data normalized to the wild-type FGF2 at 22 °C (mean ± SEM; *t-test*, *, ^#^*p* ≤ 0.05, **, ^##^*p* ≤ 0.01; *n* = 3; statistical analysis in the absence (*) or presence (^#^) of heparin). (**B**) RCS cells were treated with fresh or pre-incubated (60 °C/1 h) FGF2 variants for 10, 30 and 60 min. Phosphorylated (p) ERKs and Gab1 were detected by western blot; total ERKs and Gab1 served as loading controls. Densitometric analyses of p-protein signal are shown, data relative to a 10-min treatment of fresh FGF2 (mean ± SEM; multiple *t-test*, ***p ≤ 0.001; n, number of independent experiments)

Heparan sulfate (HS) and heparin are known to be crucial for FGF activity due to their involvement in the formation of the FGF: FGFR signaling complex on the cell surface [60] and stabilization of the ligand [29, 61, 62]. To test whether the enhanced thermodynamic stability of multiple FGF2 variants could mimic the protective effects of heparin/HS, we performed an analysis examining the activity of the variants after preincubation at elevated temperatures (range from 22 °C to 80 °C) in the presence and absence of heparin (Fig. 3A, Fig. S6). RCS cells, which produce an abundant extracellular matrix rich in sulfated proteoglycans, were treated with fresh and challenged (heated) FGF2 variants (20 ng/mL) and the activation (phosphorylation) of ERKs following 10-min stimulation was analyzed by western blotting. In addition, we performed incubation at elevated temperatures with excess heparin to counteract the inactivating effect of heat on FGF2 variants. As we predicted, the addition of heparin significantly increased temperature resistance for both the wild-type protein (50% of protein’s activity shifted from 44.5 °C to 64.6 °C) and the stable variant 8XC2 (from 63.8 °C to 74.2 °C). A similar effect was observed after 30 min of stimulation of RCS cells. Interestingly, the stable 8XC2 variant (incubated without the addition of heparin) showed biological activity like the wild-type protein in the presence of heparin. This shows that the increased thermodynamic stability can completely compensate for the lack of heparin in the system.

### Functional analysis of stable FGF2 variants: signaling, cell proliferation, migration, and metabolism

A detailed analysis of FGFR-dependent signaling pathway activation by the stable FGF2 variants, 8XC2 and 8XC2H1, was performed in RCS cells. In these cells, stimulation with the 8XC2 induced phosphorylation of FRS2, GAB1, ERKs, and PLCγ similar to that induced by the wild-type protein (Fig. S8A). No differences in response time and duration of phosphorylation were observed. In a subsequent experiment, both the wild-type protein and its stable form were incubated in complete culture medium at 60 °C for 1 h. The wild-type FGF2 failed to activate downstream signaling in RCS cells after this treatment, whereas FGF2 8XC2 retained its ability to activate FRS2, GAB1, ERK, and PLCγ and was not affected by the elevated temperature (Fig. S8B, Fig. 3B). Similarly, the 8XC2H1 variant maintained its biological activity after 1 h of incubation at 60 °C, confirming its thermal resistance in tissue culture medium (Fig. 3B).

**Fig. 4:**
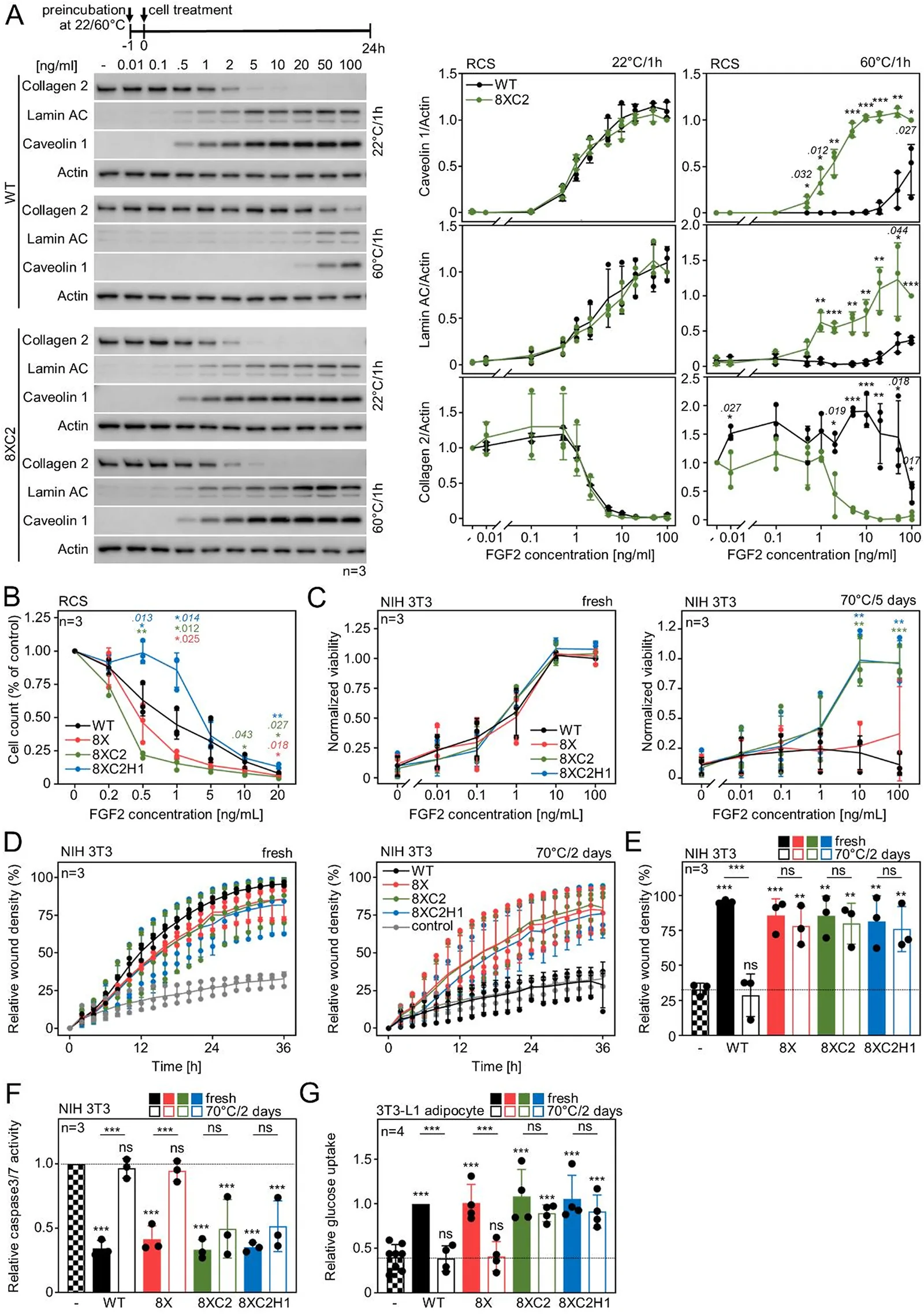
Biological activity of stable FGF2 variants. (**A**) Induction of premature senescence in RCS cells treated with FGF2 variants (0.01–100 ng/mL) for 24 h, as evidenced by upregulation of lamin A/C and caveolin 1 and degradation of ECM (collagen 2 loss). Statistical analysis was performed by *t*-test, *p ≤ 0.05, **p ≤ 0.01, ***p ≤ 0.001; *n* = 3; data are presented as mean ± SD. (**B**) Growth arrest of RCS cells induced by stable FGF2 mutants. RCS cells were cultured in complete medium and treated with various concentrations of FGF2 variants (from 0.5 to 20 ng/mL). After 96 h, cells were trypsinized and counted using Beckman Coulter Z2. The number of untreated cells after 96 h served here as a control (100%). Data are presented as mean ± SD; *n* = 3; *t*-test, *p ≤ 0.05, **p ≤ 0.01. (**C**) Viability of NIH 3T3 cells following stimulation with fresh stable FGF2 mutants and proteins preincubated at 70 °C for 5 days. Serum-starved cells were treated with FGF2 mutants at concentrations ranging from 0 to 100 ng/mL in the presence of 10 U/mL of heparin. After 48 h, metabolic activity of cells was assessed by AlamarBlue (resazurin). The maximum response for fresh wild-type FGF2 (100 ng/mL) served as 100%. Data are presented as mean ± SD; *n* = 3; *t*-test, **p ≤ 0.01, ***p ≤ 0.001. (**D**,** E**) Migration potential of stable multiple FGF2 variants estimated using the scratch wound-healing assay. NIH 3T3 cells were cultured in DMEM in the presence of 10 U/mL of heparin and treated with 10 ng/mL of fresh and challenged (70 °C for 2 days) FGF2 variants. After 36 h, relative wound density (E) was calculated and presented as mean ± SD; one-way ANOVA, **p ≤ 0.01, ***p ≤ 0.001; *n* = 3. Graphs show the overall kinetics of scratch healing (**D**), as well as the level of overgrowth at 36 h of the experiment (**E**). (**F**) NIH 3T3 cells were serum-starved for 24 h and then incubated for 24 h with fresh or challenged (70 °C for 2 days) variants of FGF2 (10 ng/mL) in the presence of 10 U/mL heparin. Cell viability and caspase-3/7 activity were measured using the ApoLive-Glo Multiplex Assay (Promega) according to the manufacturer’s protocol. The ratio of caspase-3/7 activity to cell viability was normalized against untreated cells. Mean ± SD; one-way ANOVA, ***p ≤ 0.001; *n* = 3. (**G**) Effect of stable multiple FGF2 mutants on glucose uptake by NIH 3T3-L1 adipocytes. Cells were cultured for 18 h in glucose-free medium supplemented with 10 ng/mL fresh or challenged (70 °C for 2 days) FGF2 variants in the presence of 10 U/mL of heparin. After this time, glucose uptake was measured according to the manufacturer’s protocol. Data were normalized to the maximum response for fresh wild-type FGF2 and presented as mean ± SD; one-way ANOVA, ***p ≤ 0.001; *n* = 4

In addition to the short-term cellular response, we analyzed how long-term stimulation with stable FGF2 variants affects cell fate. RCS cells are characterized by two functional long-term responses to FGF proteins, which are extracellular matrix degradation and growth arrest [58]. Extracellular matrix degradation induced by FGF2 variants was manifested by a decrease of collagen 2 level, while assessment of caveolin 1 and lamin A/C levels was used to monitor senescence progression [58, 63, 64]. Protein levels were assessed by western blotting after 24 h of treatment with FGF2 variants at different concentrations (Fig. 4A). As with the response to short-term stimulation (Fig. S3A), no difference was observed between the wild-type protein and its stable form, 8XC2. Challenging the protein by one-hour preincubation at 60 °C deprived the wild-type protein of activity, while the 8XC2 variant maintained the ability to induce extracellular matrix degradation and stimulate senescence (Fig. 4A).

We then tested FGF2 variants for growth arrest of RCS cells in response to 4-day stimulation (Fig. 4B). Since heparin affects the stability of FGF ligands and the FGF: FGFR complex, we also tested a stable mutant with a substitution that reduces affinity for heparin, 8XC2H1. RCS cells were incubated in complete medium with various concentrations of FGF2 variants (from 0.5 to 20 ng/mL). After 96 h, the cells were trypsinized and counted. Due to the high sensitivity of RCS cells to FGF and the extended time of the experiment, we were able to observe for the first time the difference between the stable 8XC2 protein and the wild-type protein without any pre-incubation at elevated temperatures. At lower concentrations (0.5 and 1 ng/mL), the inhibition of cell growth was much stronger for 8XC2 compared to the wild-type, while for the 8X variant (less proteolytically resistant than 8XC2) the difference was observed only at the concentration of 1 ng/mL. As we expected, 8XC2H1 variant showed a weaker effect compared to other stable variants due to its reduced binding to heparin (Fig. 4B).

Next, we looked at stimulation of NIH 3T3 cell proliferation, which also requires long-term incubation of cells with FGF proteins. Cells were treated with FGF2 variants for 48 h, and then their viability was analyzed with resazurin. We found that all FGF2 proteins showed comparable mitogenicity among themselves at all concentrations tested (0.01–100 ng/mL; Fig. 4C). Only after pre-challenging (5 days at 50–70 °C) of the FGF2 variants we noticed the differences – 8XC2 and 8XC2H1 maintained their mitogenic activity, while the wild-type and 8X proteins were inactivated (Fig. 4C, right panel, Fig. S7).

In addition to its mitogenic properties, FGF2 is also involved in cell migration and exhibits anti-apoptotic activity [65, 66]. Therefore, we analyzed FGF2 variants in wound healing assays (Fig. 4D, E). For this purpose, we generated a scratch in confluent NIH 3T3 and then stimulated cells for 36 h with FGF2 variants at a concentration of 10 ng/mL. As expected, all variants behaved very similarly and presented the same recolonization kinetics. However, incubation at high temperature (2 days at 70 °C) before addition to the cells resulted in a lack of wild-type migration activity while all stable variants (8X, 8XC2, 8XC2H1) stimulated recolonization of the wound area (Fig. 4E). We then evaluated the anti-apoptotic effect of FGF2 mutants in serum-starved NIH 3T3 cells by measuring caspase-3/7 activity (Fig. 4F). The protective effects of the tested FGF2 variants were not significantly different when the “fresh” proteins were used. In contrast, treatment of the proteins with high temperature (2 days at 70 °C) resulted in loss of activity not only of the wild-type, but also of 8X mutant, with an unchanged anti-apoptotic response for the 8XC2, 8XC2H1 variants (Fig. 4F).

Moreover, we set out to test whether, like FGF1, FGF2 and its variants display metabolic activity in an in vitro adipose tissue cell model [50, 67, 68]. For this purpose, differentiated 3T3-L1 adipocytes were treated with fresh and challenged (2 days at 70 °C) FGF2 variants at a concentration of 10 ng/mL for 18 h and analyzed for glucose uptake (Fig. 4G). Fresh stable FGF2 mutants showed no metabolic activity greater than the wild-type protein. However, subjecting the proteins to thermal preincubation abolished the metabolic properties for wild-type and 8X (Fig. 4G). It is noteworthy that FGF2 stabilization raising only its denaturation temperature (8X) was not effective enough to protect the protein from the loss of some biological activities due to prolonged incubation at elevated temperatures, in contrast to the situation when stabilizing mutations were combined with substitutions extending the half-life of the protein (8XC2) (Fig. 4C, F, G).

### Translational Implications: Stable FGF2 Promotes Stem Cell Survival and Self-Renewal

**Fig. 5:**
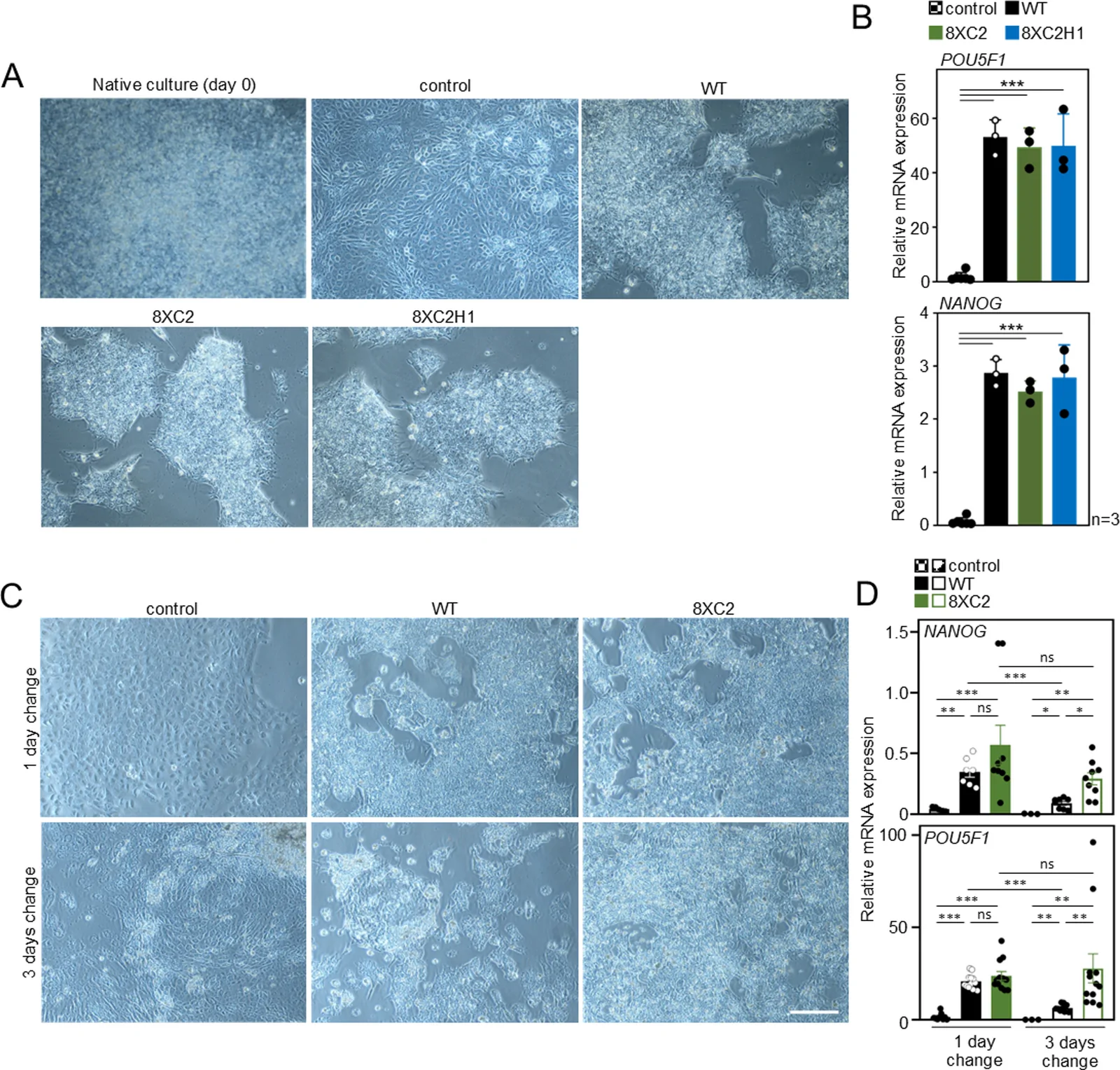
Increased stability of FGF2 prolongs maintenance of iPS cell pluripotency. (**A**) Morphology of iPS cells cultured on Matrigel in conditioned medium without FGF (control) or supplemented with FGF2 variants (wild-type, 8XC2, 8XC2H1) for three passages (10–12 days). In addition, an image of the cells on day 0 is shown. Medium was changed daily, scale bar 200 μm. (**B**) qPCR data for mRNA expression of pluripotency markers *POU5F1* and *NANOG*; mean ± SD; one-way ANOVA, ***p ≤ 0.001; *n* = 3. (**C**) Morphology of iPS cells on Matrigel in conditioned medium without FGF (control) or supplemented with FGF2 variants (wild-type, 8XC2) for five passages (30–36 days) a change of medium daily or every three days. Scale bar 200 μm. (**D**) qPCR data for mRNA expression of pluripotency markers *POU5F1*, *NANOG*; mean ± SD; Mann-Whitney test, *p ≤ 0.05, **p ≤ 0.01, ***p ≤ 0.001; *n* = 3

FGF2 is known for its key role in stem cell adhesion, self-renewal and maintenance of pluripotency [69, 70]. It appears that growth factor stability may further regulate these processes. It was shown that FGF1, which binds the same FGFRs as FGF2, but is a less stable protein, did not maintain the pluripotency of embryonic stem cells. However, when stabilized by point mutations, it exhibited an effect similar to FGF2 [71]. To test the effect of stable FGF2 variants (8XC2 and 8XC2H1) on stem cells, we used human iPSC (cl.4 line) [51] and verified their morphology (Fig. 5A and Fig. S11A) and *POU5F1* and *NANOG* mRNA expression (Fig. 5B). Both stable variants, like the wild-type protein, resulted in the same size and shape of cells, maintaining their morphology and proliferation. In contrast, in the FGF2-unstimulated culture, clear signs of differentiation could be observed in morphology of the cells from the second passage (Fig. 5A). Consistent with the observed morphology, FGF2-stimulated cells showed high expression of the pluripotency marker genes, *POU5F1* and *NANOG* (Fig. 5B). We then analyzed how frequent FGF2 re-supplementation is necessary for long-term stem cell propagation. For this purpose, human iPSCs were propagated with wild-type FGF2 and 8XC2 FGF2, changing the medium daily or every three days. We observed that with every three-day supplementation, both morphology and mRNA expression of *POU5F1* and *NANOG* changed for the wild-type FGF2 (compared to daily medium change), indicating a loss of its biological activity, while 8XC2 FGF2 retained its properties without the need to add the protein daily (Fig. 5C, D, Fig. S11B). Finally, we investigated whether the prolonged bioactivity of stable FGF2 variants might have impaired the differentiation process. hiPS cl.4 cells were cultured in the presence of 100 ng/mL FGF2 variants for 9 days, after which FGF2 proteins were removed, and the cells were allowed to differentiate spontaneously for up to 12 days. qPCR analysis of the expression of germ layer markers PAX6 (ectoderm), TBX6 (mesoderm), and FOXA (endoderm) revealed normal differentiation induction (Fig. S12). These data indicate that incubation with stable FGF2 variants does not prolong the time required to exit the pluripotent state.

## Discussion

The intrinsic instability of diverse proteins remains a fundamental barrier to their widespread clinical and industrial application. In this study, we overcame the significant thermal sensitivity of FGF2 by engineering highly stable variants that circumvent the typical compromise between stability and function. By combining a consensus approach with structure-based rational mutagenesis, we achieved a stabilization effect (over 27 °C) that effectively eliminates thermal denaturation as a limiting factor under physiological and even extreme conditions.

While the therapeutic landscape includes successful protein drugs stabilized via extrinsic modifications, such as PEGylation (e.g., Pegloticase) [72] or Fc-fusion (e.g., TNFR-Fc) [73], these strategies often increase hydrodynamic size, potentially hindering tissue penetration and receptor accessibility [74, 75]. In contrast, our approach focused on intrinsic sequence optimization. Similar strategies have yielded clinically approved agents like Palifermin (truncated FGF7) [76] or drug candidates like engineered FGF1 [50, 77] and disulfide-stabilized FGF21 [78]. However, the magnitude of the effect we report here, maintaining full mitogenic competence after 5 days at 70 °C, far exceeds typical engineering outcomes. This “hyper-stability” suggests that the engineered FGF2 scaffold has reached a thermodynamic potential comparable to proteins from thermophilic organisms, preventing the unfolding events that typically initiate aggregation and proteolytic clearance [79, 80].

Here, we show the direct translation of thermodynamic stability into prolonged biological activity. Wild-type FGF2 has a half-life of mere hours in culture media due to rapid degradation [29, 81]. Our variants (specifically 8XC2H1) remained active for days in the presence of adipocytes, a proteolytic-rich environment that mimics metabolic tissue. This resistance likely arises from structural rigidification via optimized secondary structure elements and the introduction of stabilizing hydrogen bonds, together with the elimination of reactive surface cysteines (C78, C96). Eliminating these thiols minimizes oxidative aggregation by altering surface thiol chemistry and contributes to resistance against proteolytic degradation [54, 82, 83].

Furthermore, we introduced a strategic functional modification – reduced heparin affinity. In canonical FGF signaling, interactions with heparan sulfate proteoglycans, while protecting against thermal and proteolytic degradation [29], sequester the growth factor in the extracellular matrix (ECM), limiting its diffusion and bioavailability [81, 84]. A key achievement of our strategy was to substitute this extrinsic stabilization with intrinsic structural optimization. By developing a hyper-stable scaffold, we rendered the protective role of heparin redundant, allowing us to introduce the K128D substitution. This mutation reduces affinity for heparin to “uncouple” ECM binding from receptor activation [85]. Consequently, our variants are designed to evade ECM accumulation, potentially improving pharmacokinetics and tissue distribution while maintaining full biological potency. Future studies should determine whether compound 8XC2H1 exhibits improved distribution and exposure in vivo due to its reduced affinity for proteoglycans on the cell surface.

Our findings represent a significant advancement over previous stabilization efforts, including the FGF2-G3 variant developed by Dvorak et al. [31]. While we incorporated specific validated substitutions (R31L, T121P) and targeted similar positions (Glu54, His59), our consensus strategy led to distinct choices, such as the H59Y mutation and serine substitutions for surface cysteines (C78S, C96S) rather than tyrosines. In a direct comparison performed in our laboratory, our lead variant (8XC2H1) exhibited a denaturation temperature approximately 7 °C higher than the G3 protein (*ΔT*_*m*_>27 °C vs. *ΔT*_*m*_≈20 °C) (Table S10). Functionally, this translated into higher biological potency. Moreover, we optimized the pharmacokinetic profile by screening eight candidate mutations targeting heparin binding sites, including positions analyzed by Okada et al. [86]. We selected the K128D substitution, which reduces heparin affinity while concurrently contributing to thermal stability. This design feature confers a distinct therapeutic advantage over the G3 variant; by dampening matrix sequestration, the 8XC2H1 variant is predicted to achieve superior biodistribution and sustained effective concentrations in the bloodstream.

The practical impact of this hyper-stability is most evident in stem cell culture. While wild-type FGF2 requires daily supplementation to prevent spontaneous differentiation, our 8XC2 variant sustained high expression of pluripotency markers (POU5F1 and NANOG) in iPSCs with a significantly reduced dosing frequency (every 3 days) [51]. This extended functional half-life directly addresses the cost and labor constraints of large-scale stem cell manufacturing.

While the enhanced stability and prolonged activity of engineered FGF2 variants offer therapeutic advantages, the potential for increased mitogenic and pro-angiogenic effects, particularly concerning tumor progression, warrants careful consideration. Studies have shown that FGF2 can accelerate early tumor development and potent vascularization [87], and even influence macrophage polarization and tumor immunity [88]. A highly stable variant, by maintaining its biological activity for extended periods, could theoretically pose a greater risk of sustained stimulation of cancer cell proliferation or other undesirable mitogenic effects compared to wild-type FGF2, which is rapidly degraded. However, it is also important to note that enhanced stability can reduce FGF2’s dependence on heparin for FGFR signaling [32], which might alter its contextual activity. Future studies will need to meticulously evaluate the in vivo oncogenic potential of these highly stable FGF2 variants in appropriate preclinical models to ensure their safe translation into clinical applications.

## Conclusions

In summary, we have developed a class of highly stable, proteolytically resistant FGF2 variants that overcome the intrinsic limitations of the natural growth factor. By combining extreme thermodynamic stability with optimized matrix interactions, these molecules offer an exceptional solution for cost-effective stem cell expansion and next-generation regenerative therapies requiring sustained signaling in harsh physiological microenvironments.

## Supplementary Information

Below is the link to the electronic supplementary material.

Supplementary Material 1

Supplementary Material 2

## Data Availability

All data generated or analyzed in the study are available from the corresponding authors on reasonable request.
