# Supplementary material for: Structure–function relationship in FGF signaling: rational design of highly stable FGF2 variants and their effect on cell growth, metabolism, and survival
Source: J Biol Eng. 2026 Jul 1;20:140. doi: 10.1186/s13036-026-00723-z (PMC13595800; doi:10.1186/s13036-026-00723-z)

Full uncropped Gels and Blots images - MANUSCRIPT

Figure 1E

wild-type

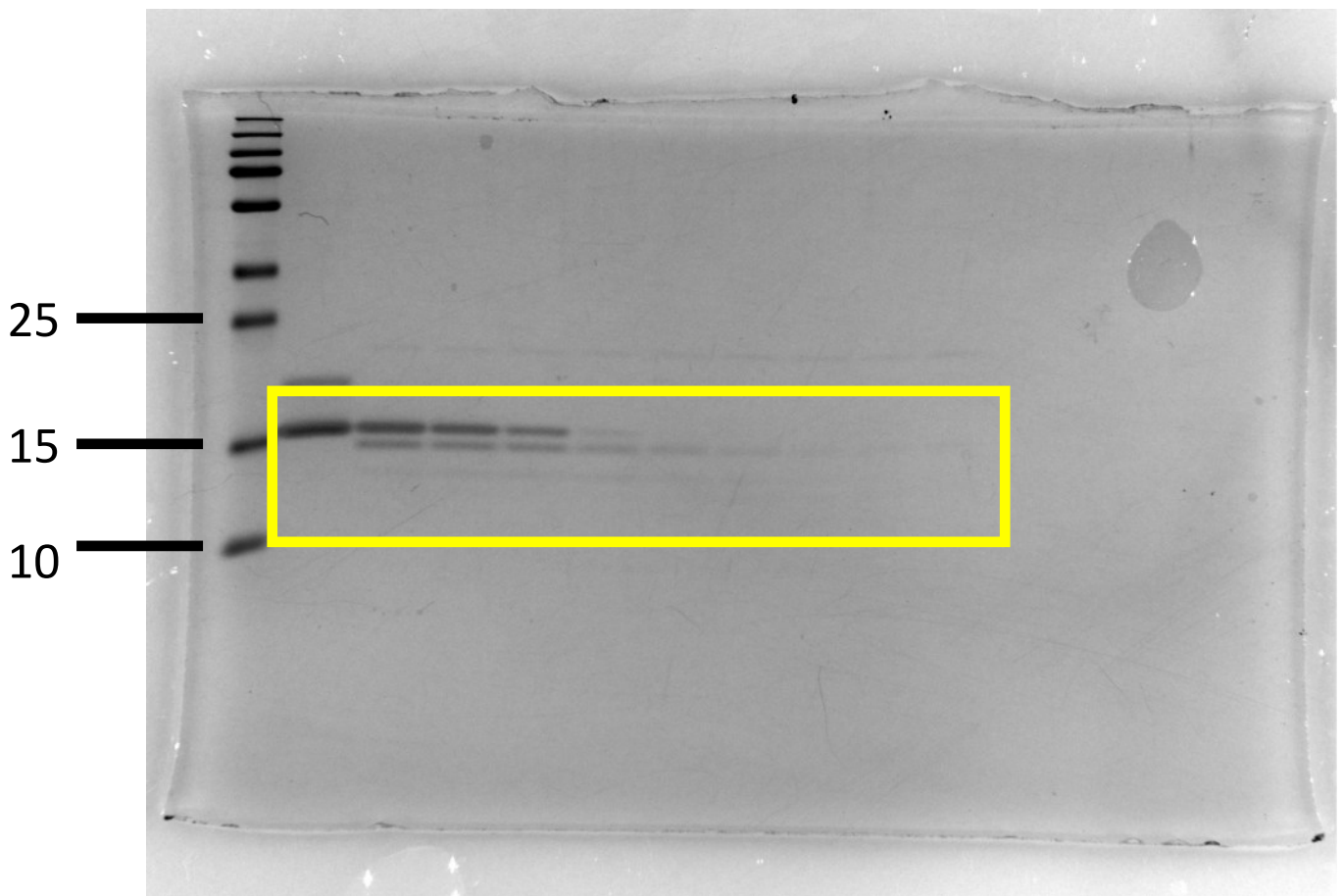

8X

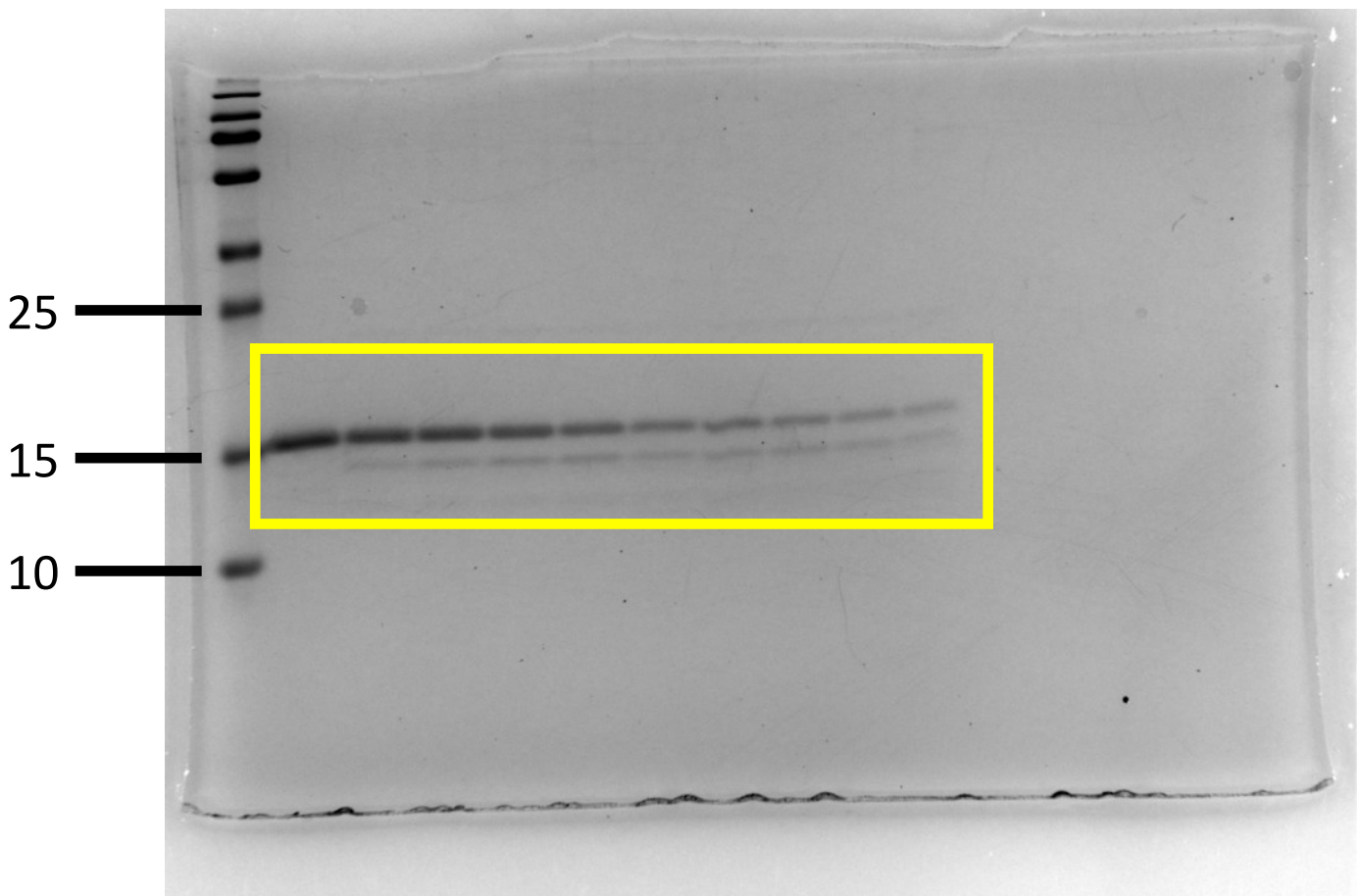

8XC2

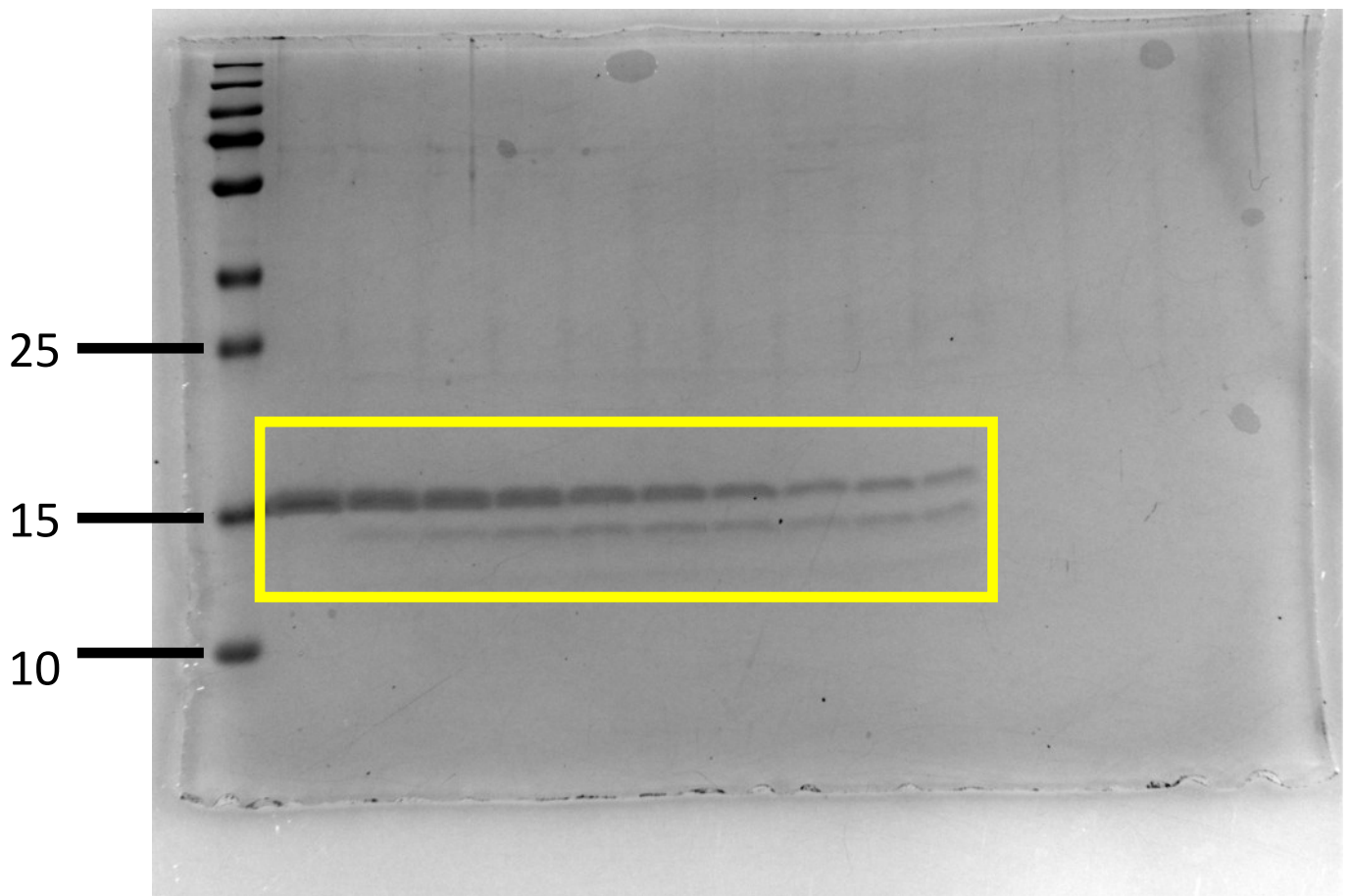

8XC2H1

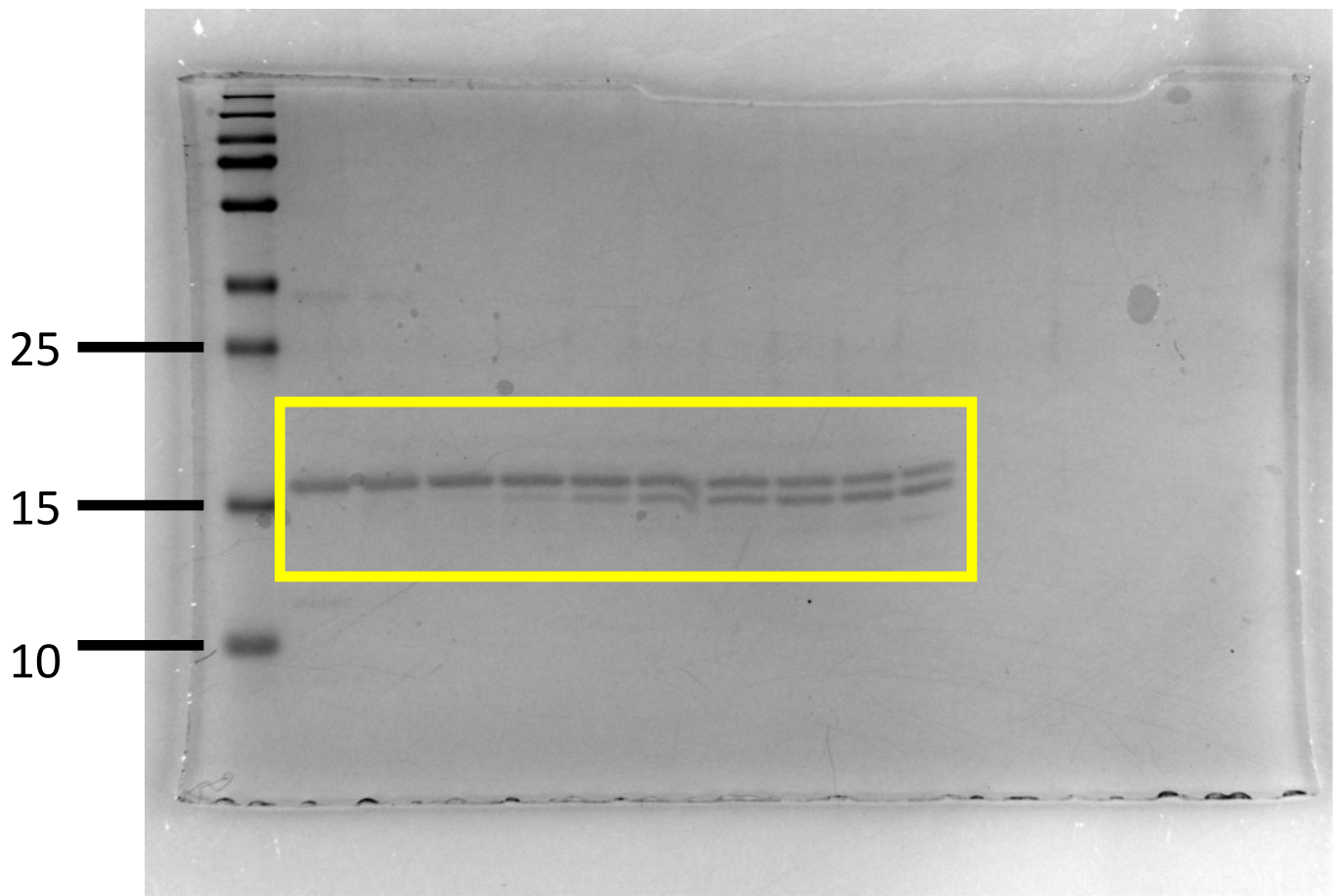

Figure 1F

pFGFR

100

70

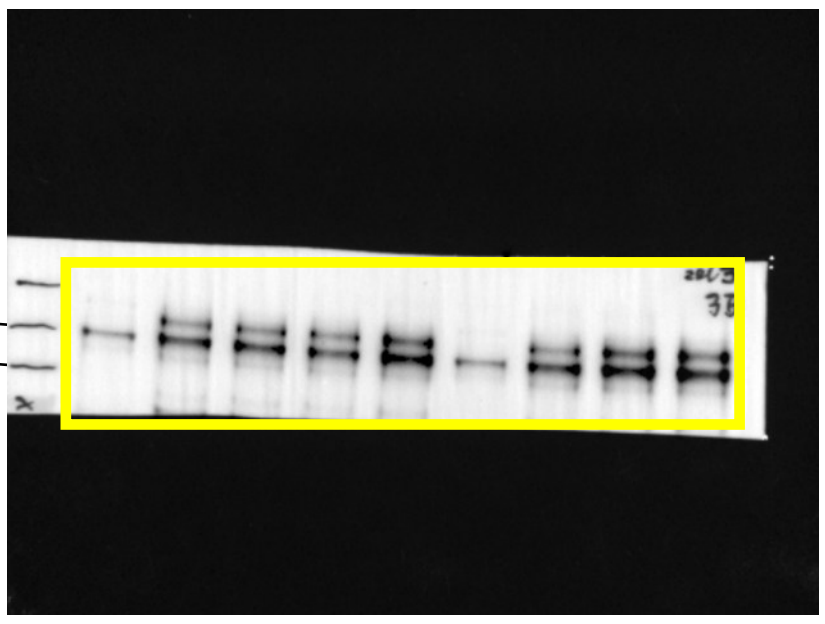

pERKs

55

35

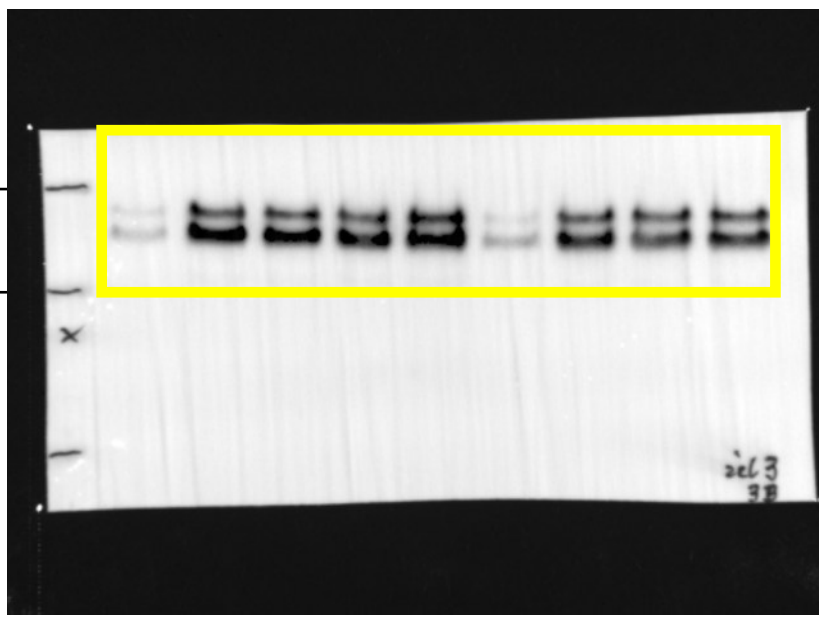

Tubulin

55

35

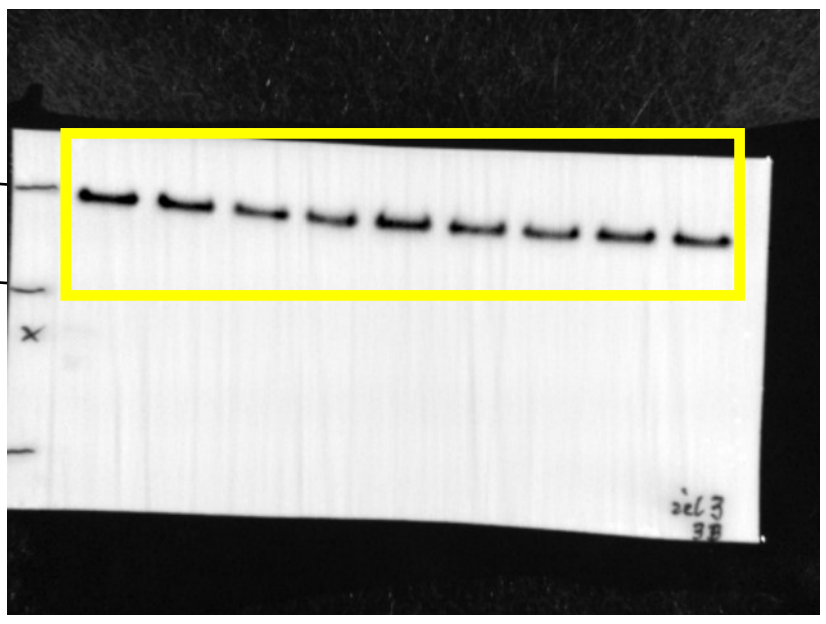

Figure 1G

pFGFR

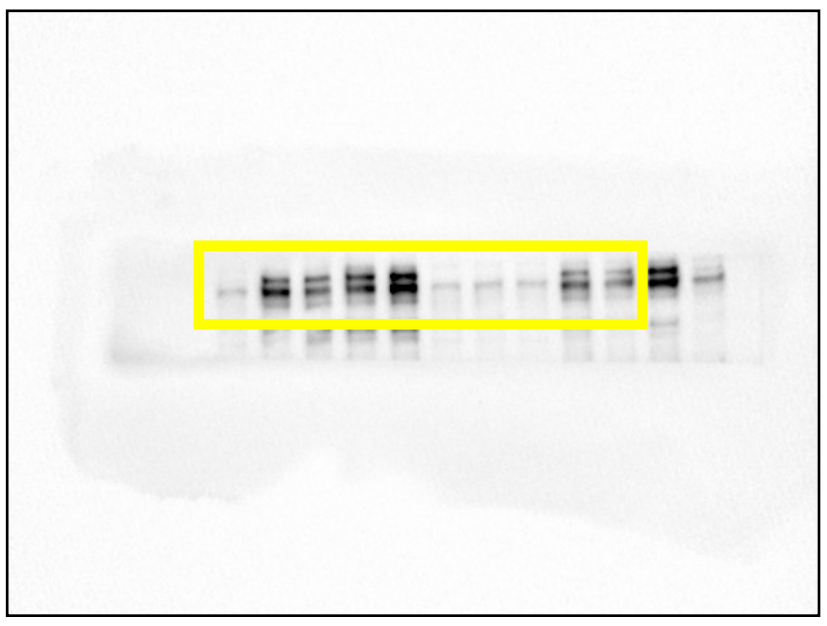

pERKs

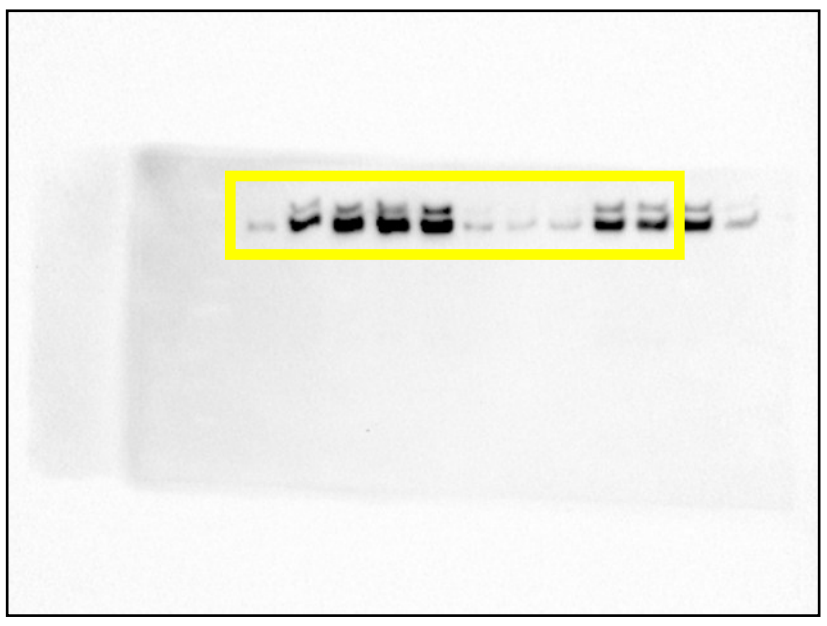

Tubulin

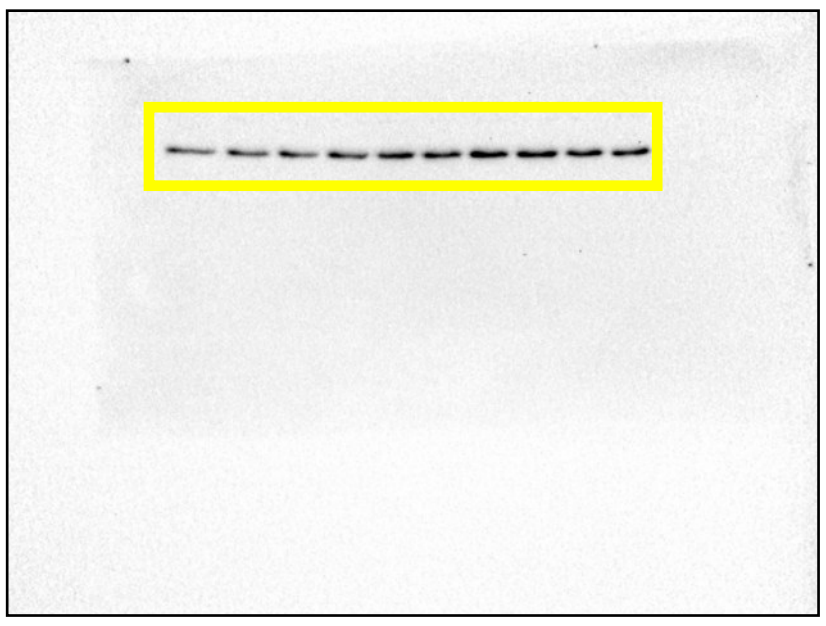

Figure 2B

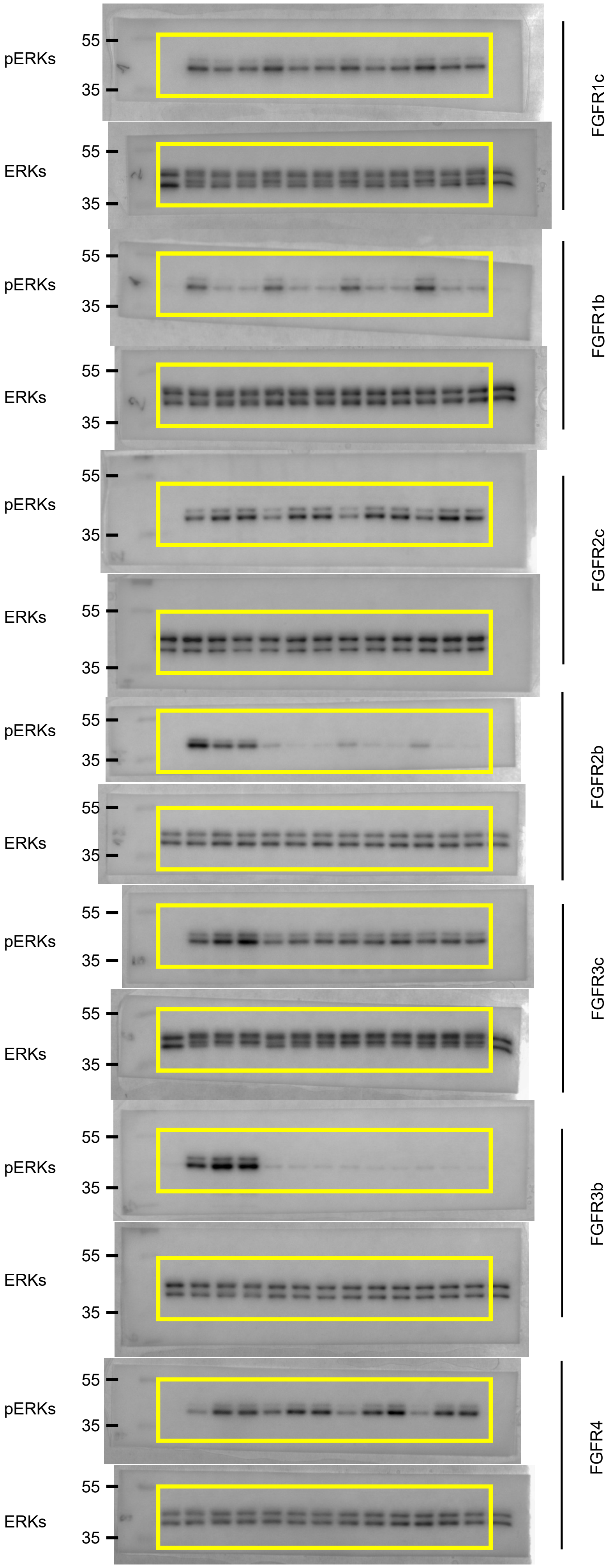

pERKs
ERKs
pERKs
ERKs
pERKs
ERKs
pERKs
ERKs
pERKs
ERKs
pERKs
ERKs
pERKs
ERKs
55
35
FGFR1c
FGFR1b
FGFR2c
FGFR2b
FGFR3c
FGFR3b
FGFR4

Figure 3A

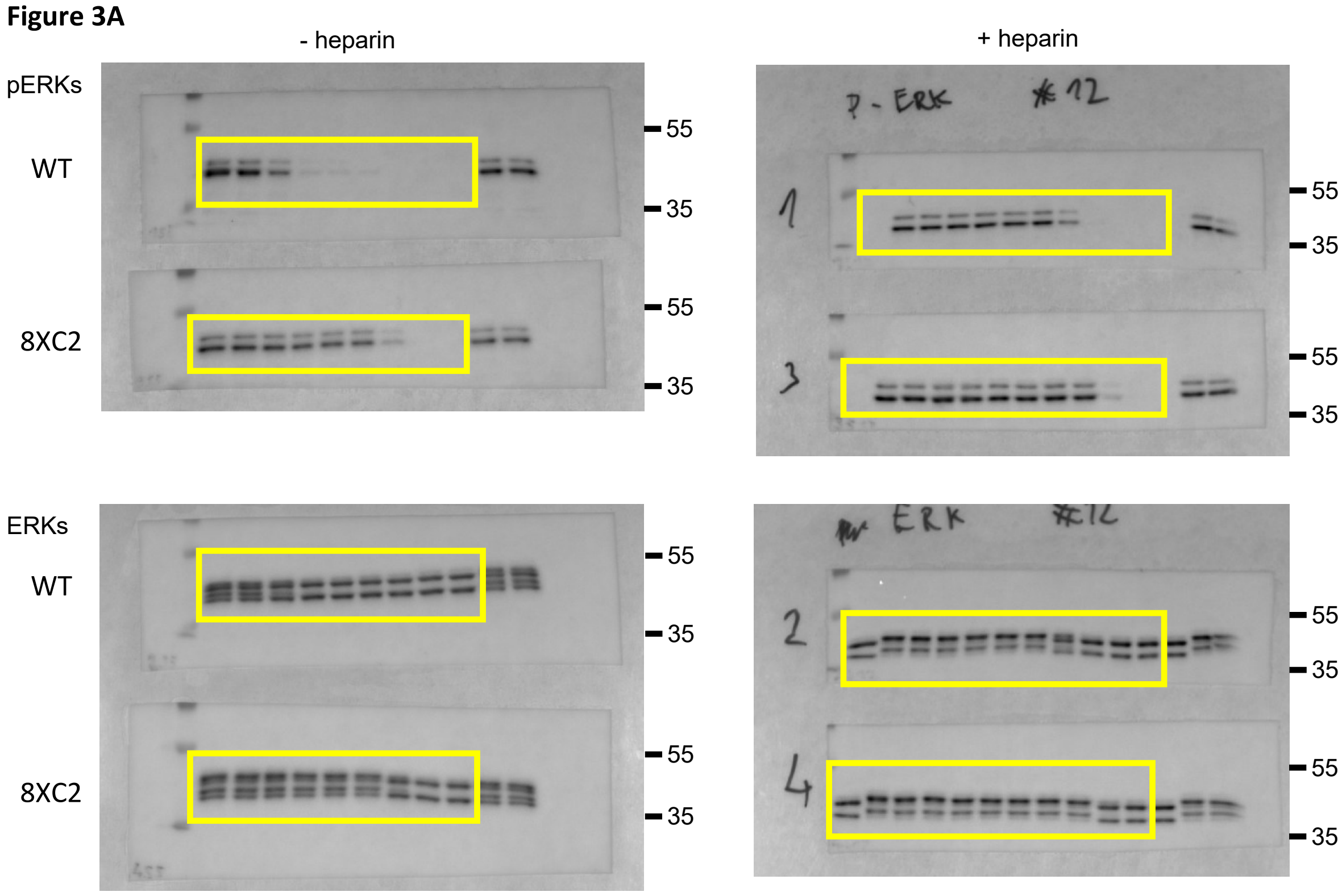

- heparin
+ heparin
pERKs
WT
8XC2
ERKs
WT
8XC2
55
35
p-ERK
ERK
1
2
3
4

Figure 3B + C

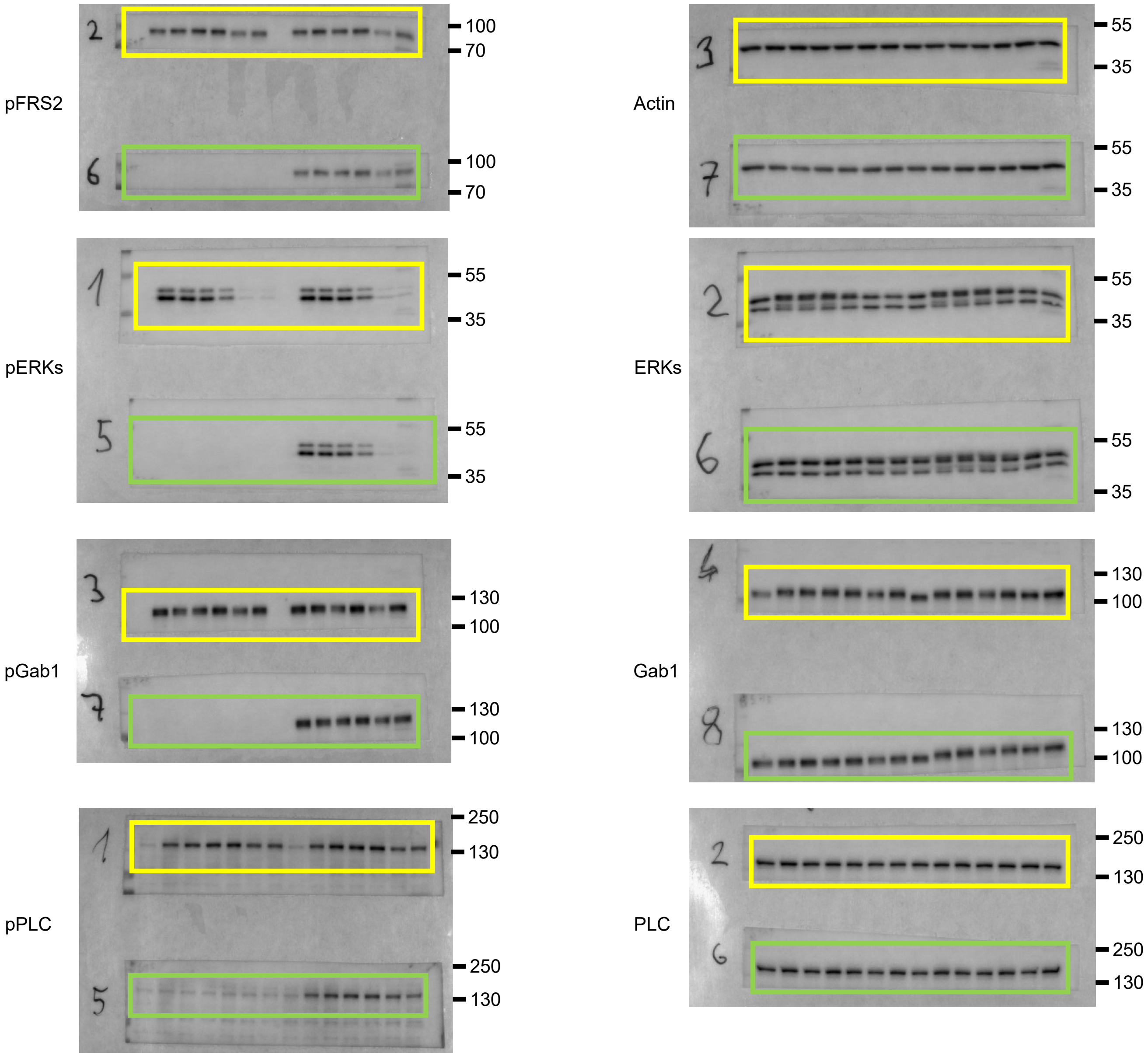
pFRS2
100
70
100
70
Actin
55
35
55
35
pERKs
55
35
55
35
ERKs
55
35
55
35
pGab1
130
100
130
100
Gab1
130
100
130
100
pPLC
250
130
250
130
PLC
250
130
250
130

Figure 4A

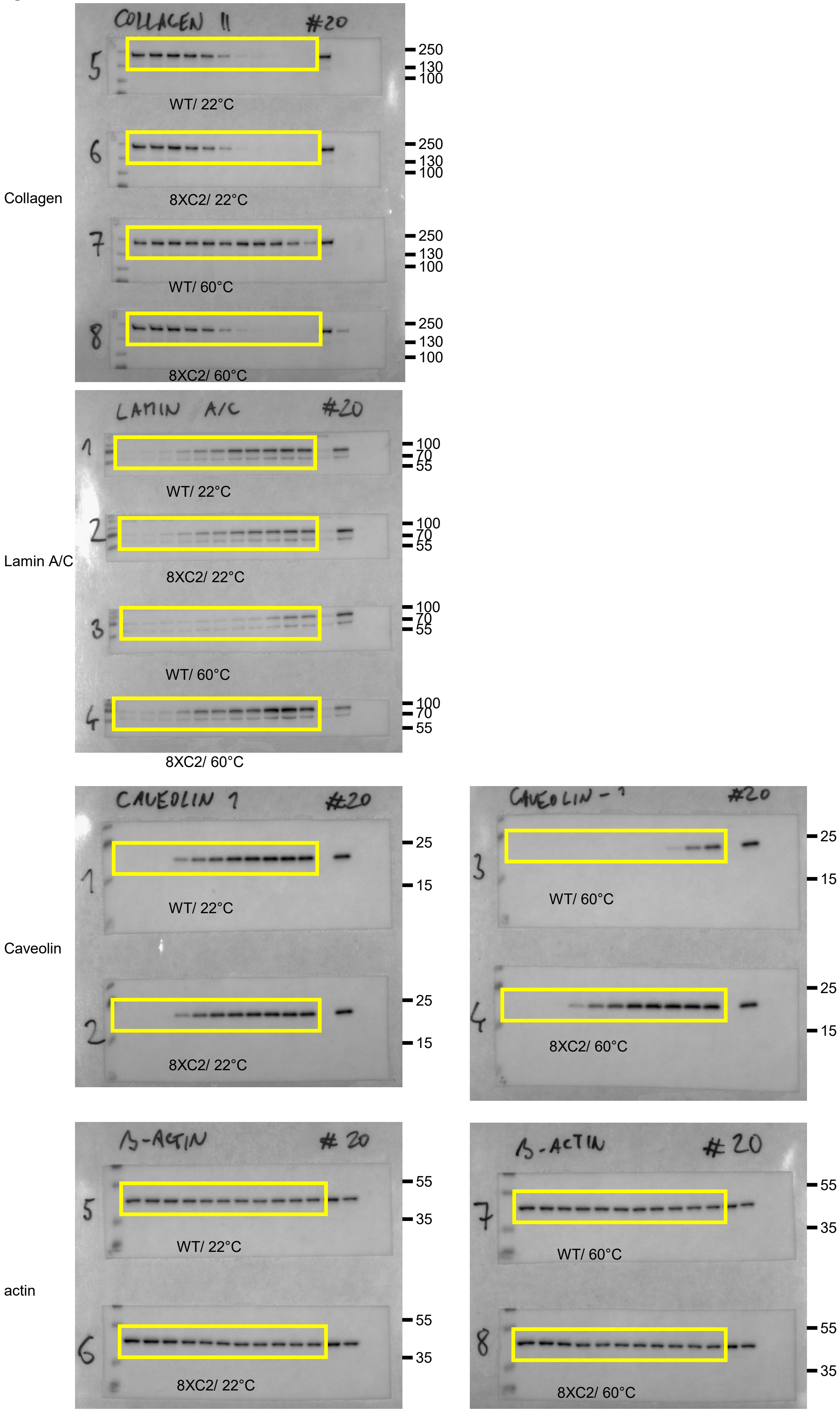
Collagen
WT/ 22°C
8XC2/ 22°C
WT/ 60°C
8XC2/ 60°C
250
130
100
Lamin A/C
WT/ 22°C
8XC2/ 22°C
WT/ 60°C
8XC2/ 60°C
100
70
55
Caveolin
WT/ 22°C
8XC2/ 22°C
WT/ 60°C
8XC2/ 60°C
25
15
actin
WT/ 22°C
8XC2/ 22°C
WT/ 60°C
8XC2/ 60°C
55
35

Supplementary Figure 1B

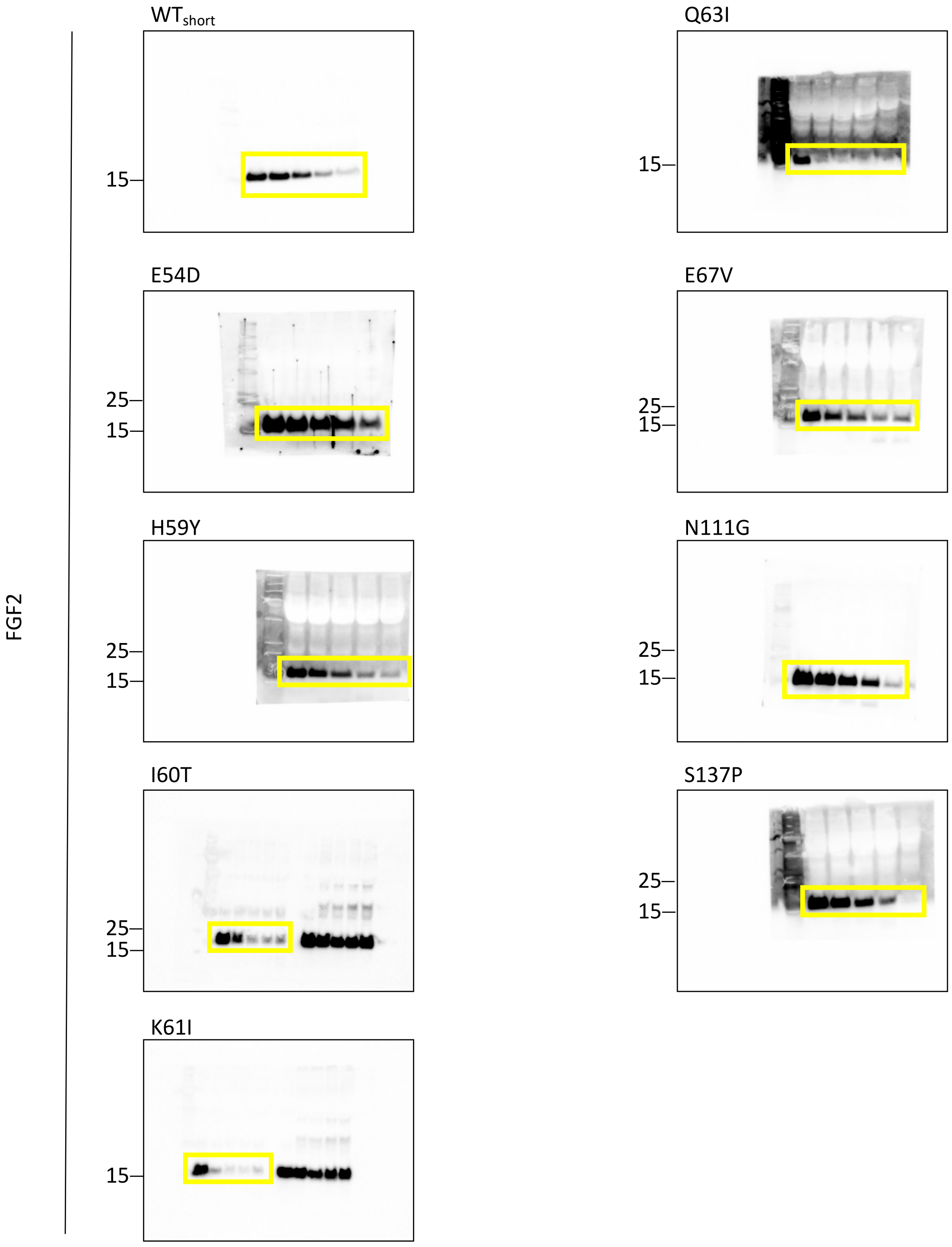

Supplementary Figure 1C

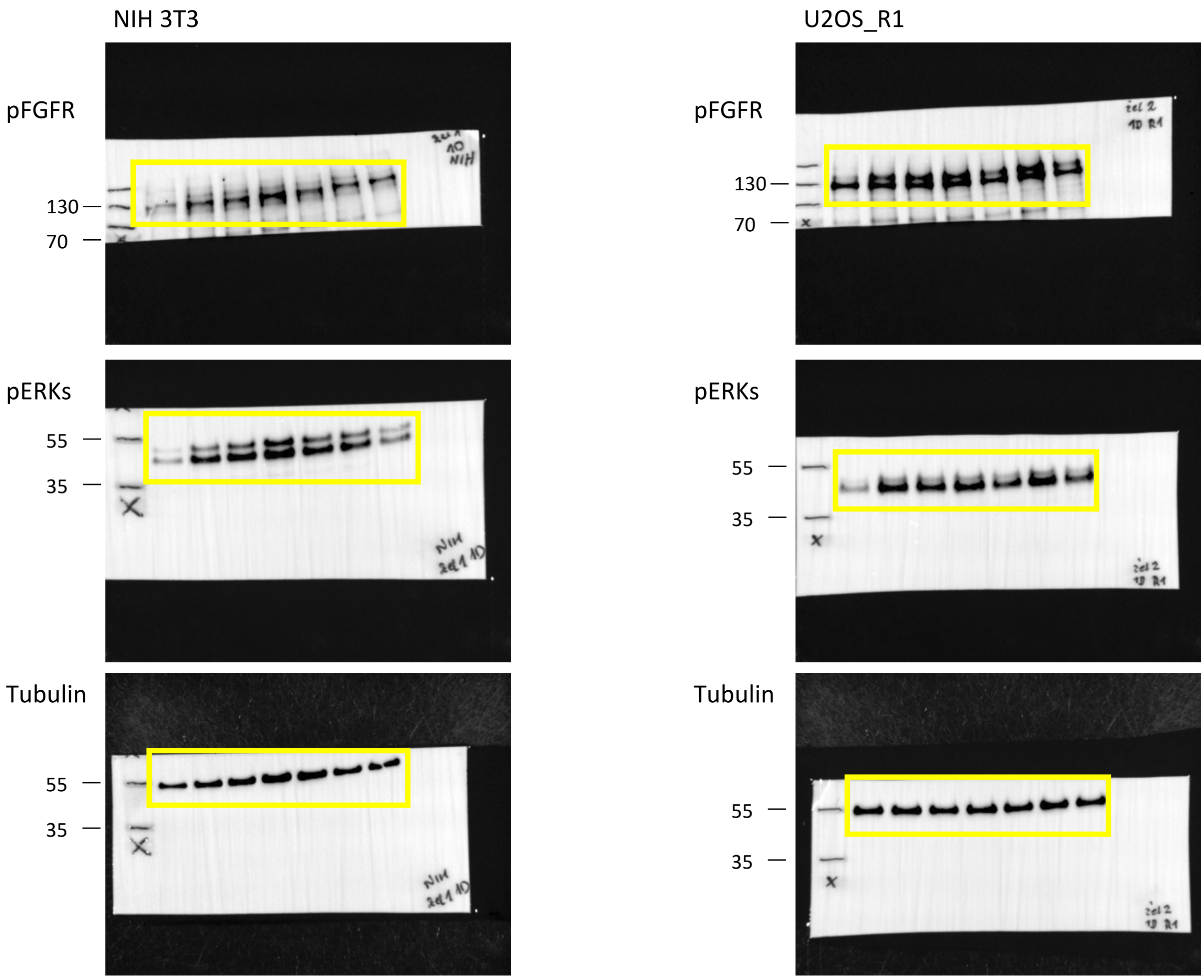

Supplementary Figure 2

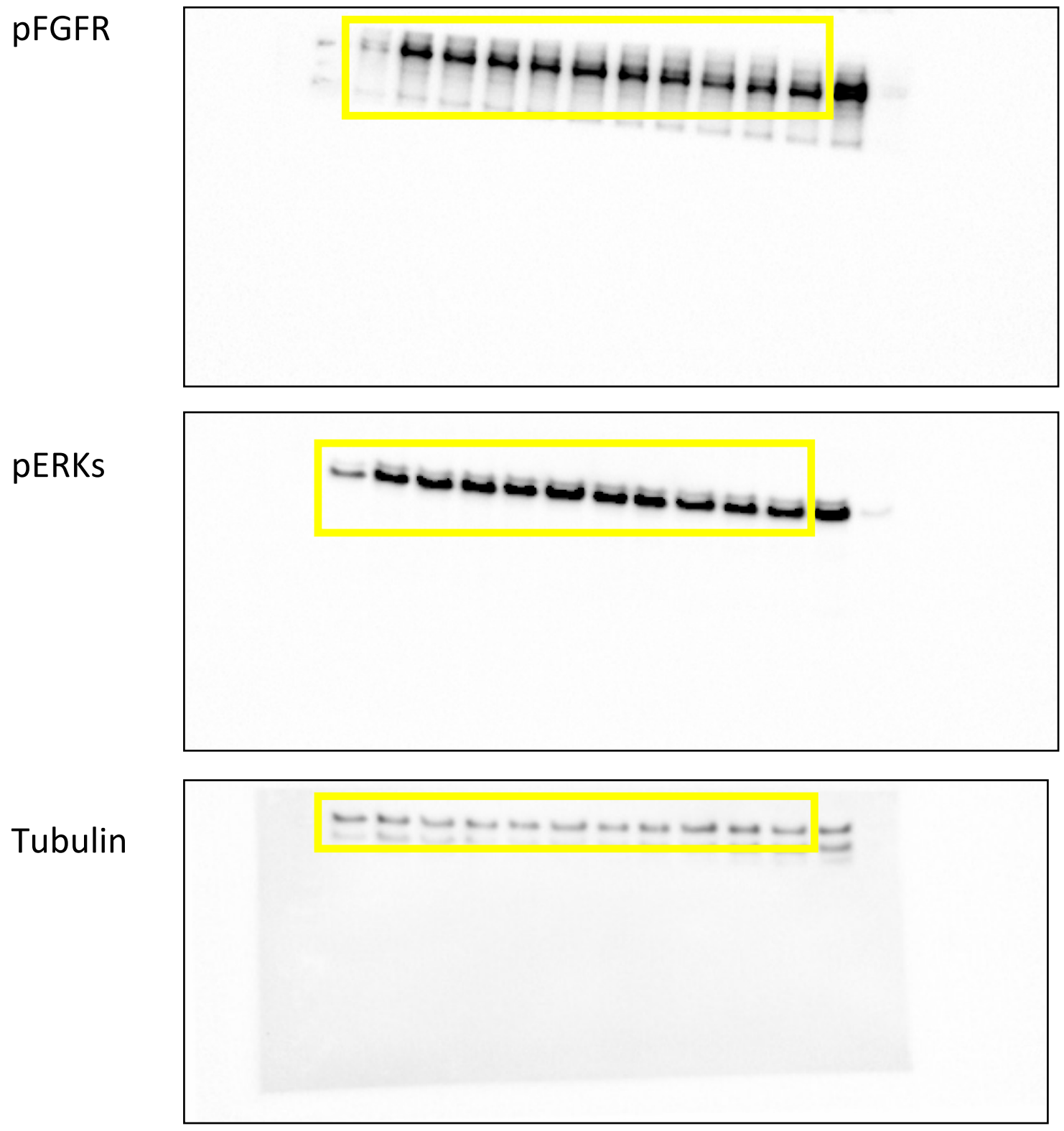

Supplementary Figure 3B

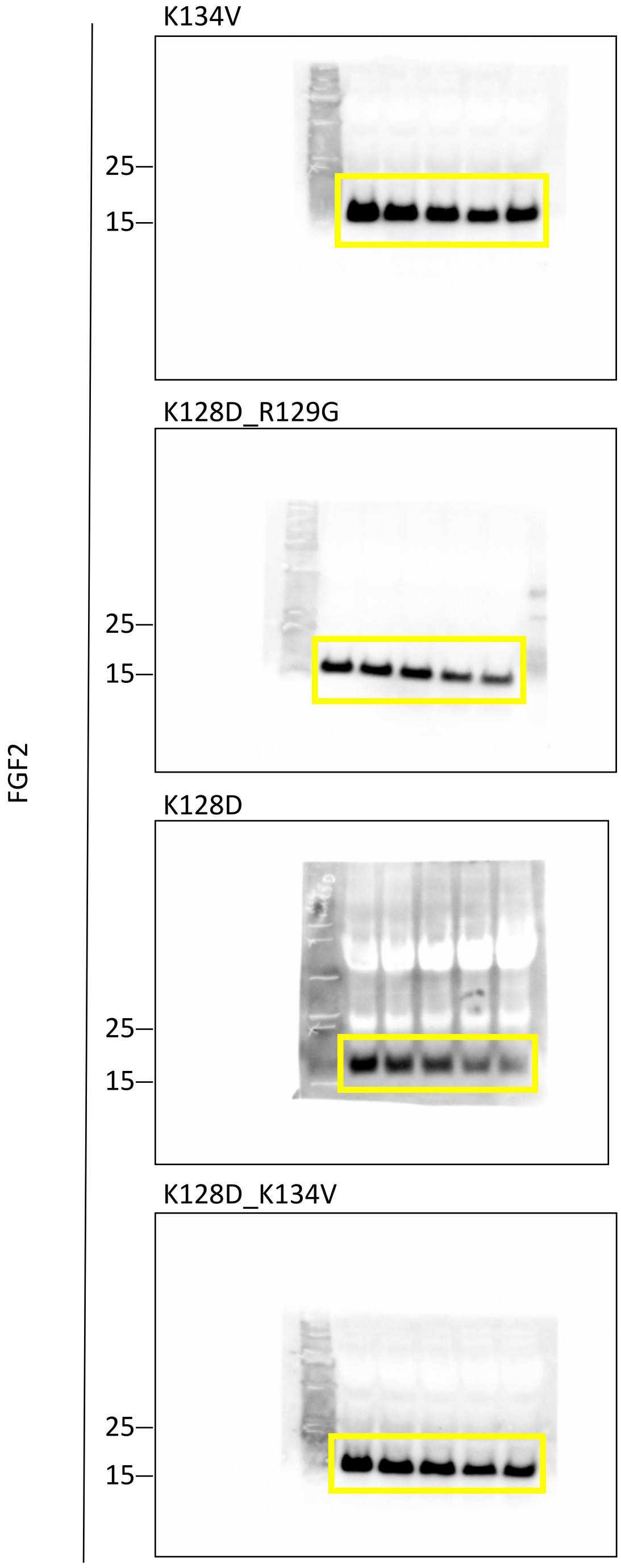

Supplementary Figure 3C

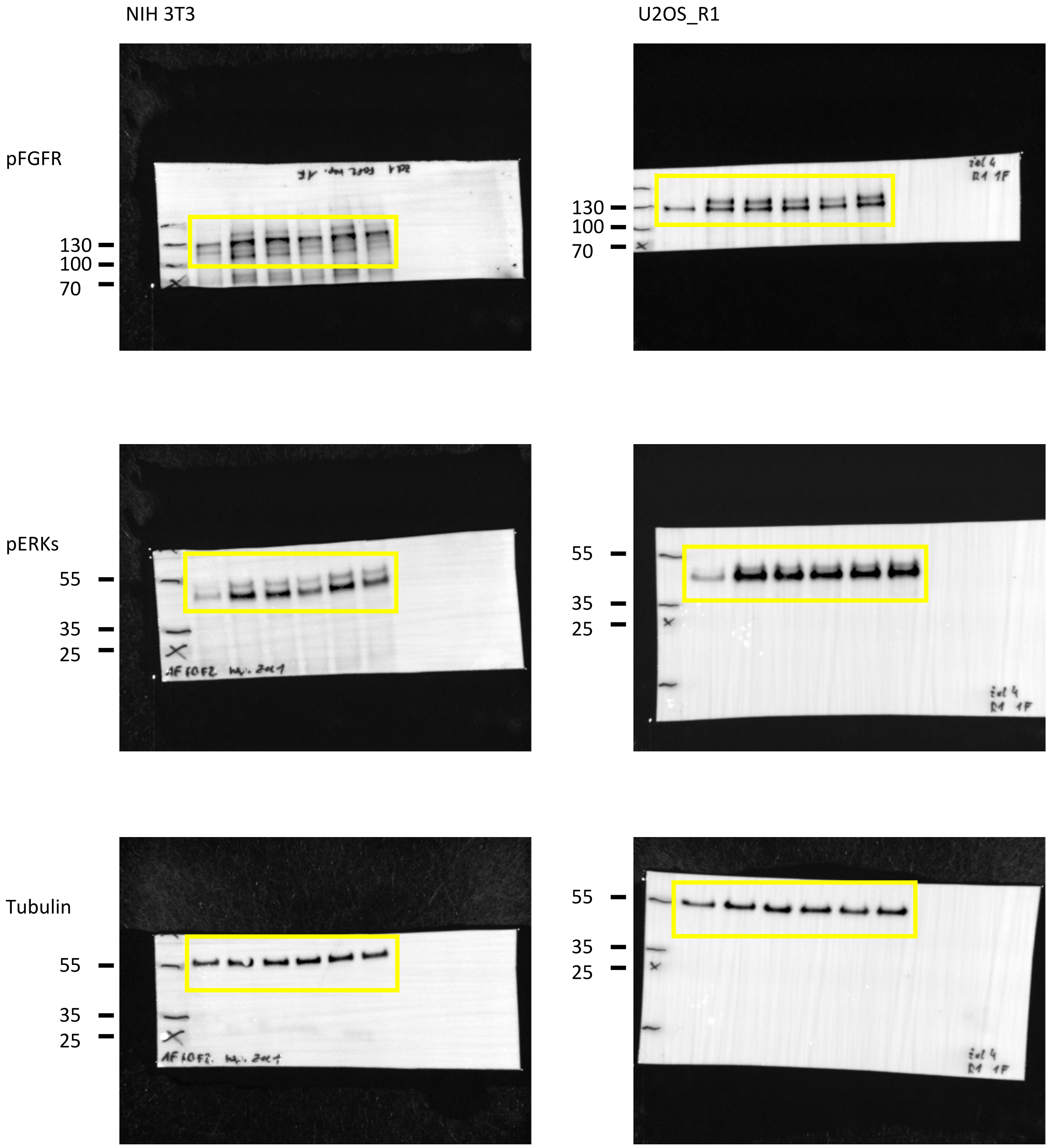

Supplementary Figure 4

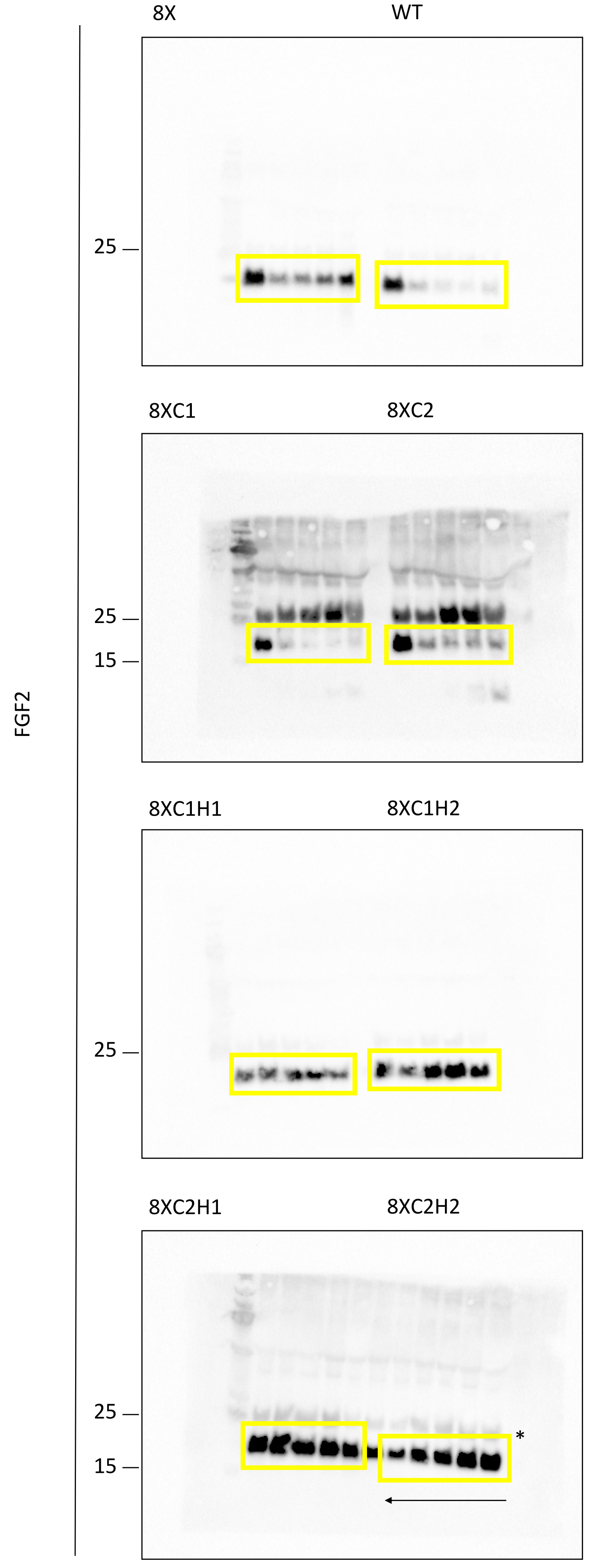

*Samples loaded in reverse order

Supplementary Figure 5

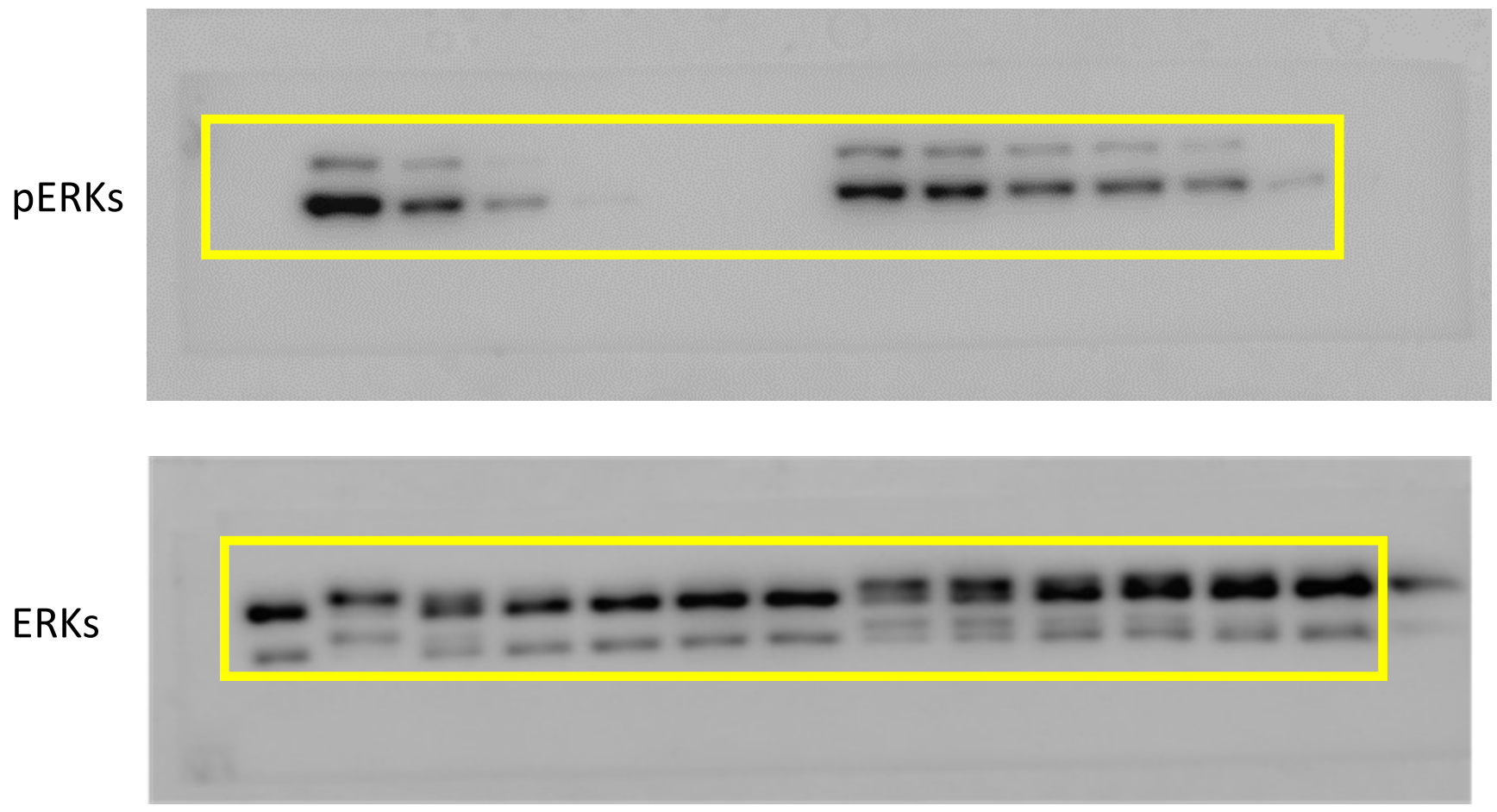

Supplementary Figure 6

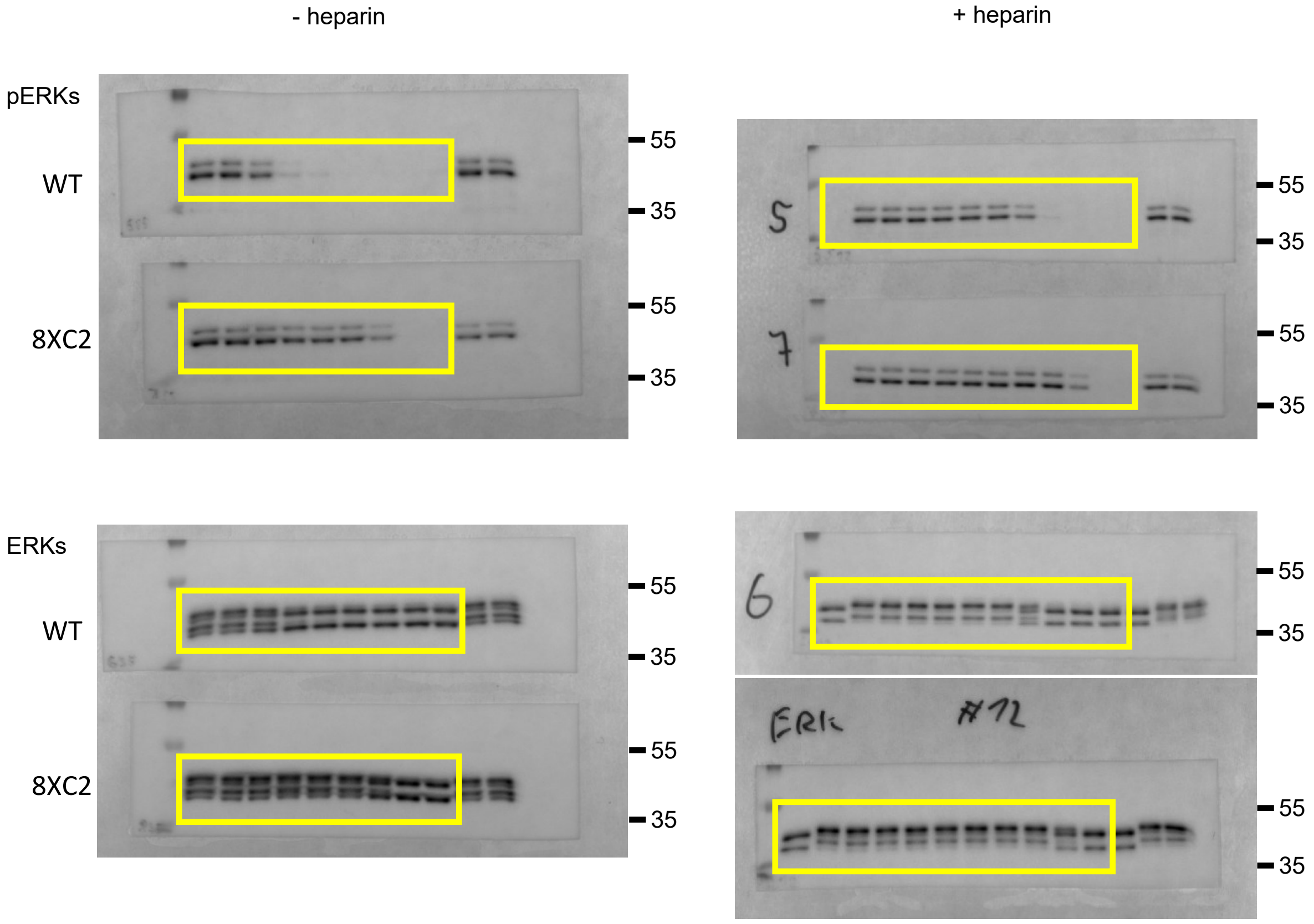

Supplement: Supplementary file 1 [file 13036_2026_723_MOESM1_ESM.pdf]
