# Supplementary material for: Structure–function relationship in FGF signaling: rational design of highly stable FGF2 variants and their effect on cell growth, metabolism, and survival
Source: J Biol Eng. 2026 Jul 1;20:140. doi: 10.1186/s13036-026-00723-z (PMC13595800; doi:10.1186/s13036-026-00723-z)
Supplement: Supplementary file 2 [file 13036_2026_723_MOESM2_ESM.pdf]

SUPPLEMENTARY MATERIALS

Supplementary Figure 1

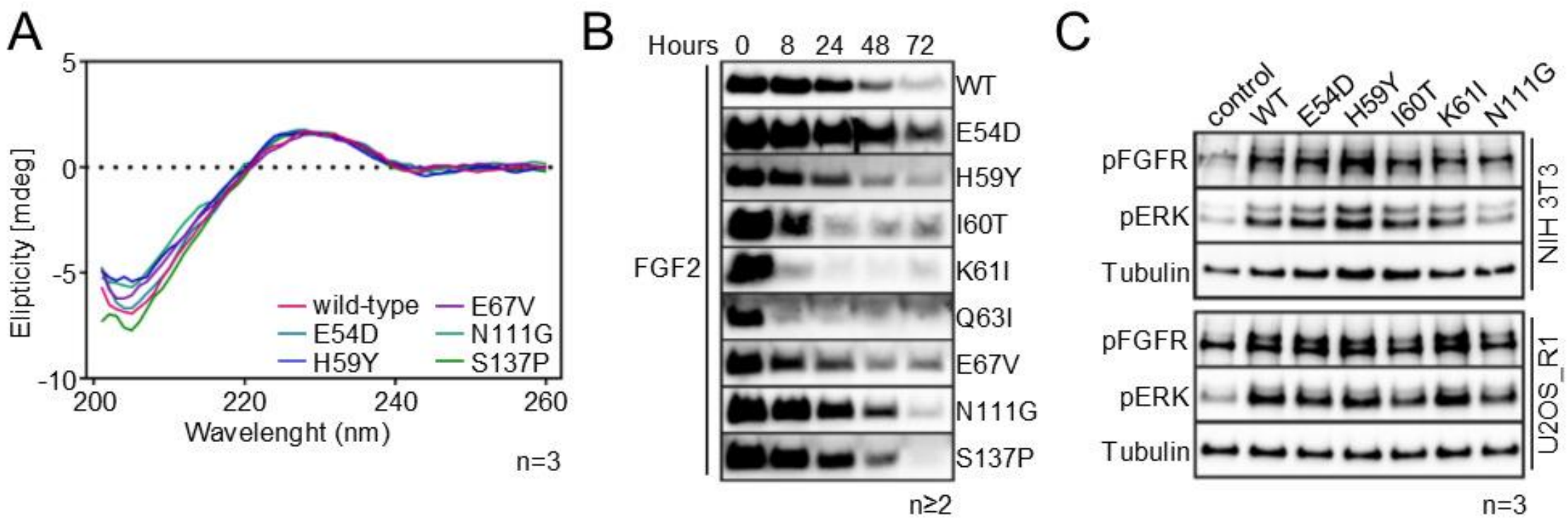

Supplementary Figure 1. Characteristics of single FGF2 variants with increased stability. (**A**) CD spectra of FGF2 variants between 200 nm and 260 nm; (n=3). (**B**) Western blot analysis of degradation of FGF2 variants with enhanced thermal stability in NIH 3T3-conditioned medium over time. 1 µg of protein was incubated in completed NIH 3T3-conditioned medium over the cells, and at the indicated time points, samples were collected and subjected to SDS-PAGE followed by western blot with anti-FGF2 antibody; (n≥2). (**C**) Activation of downstream signaling by single FGF2 variants with enhanced thermal stability in NIH 3T3 and U2OS_R1 cell lines. Cells starved overnight were treated with protein at a concentration of 10 ng/mL. After 15 min, cell lysates were collected and subjected to SDS-PAGE followed by western blot analysis. Phosphorylation analysis of FGFR and ERKs proteins was used to confirm the biological activity of the obtained variants. Tubulin served as a loading control; (n=3).

Supplementary Figure 2

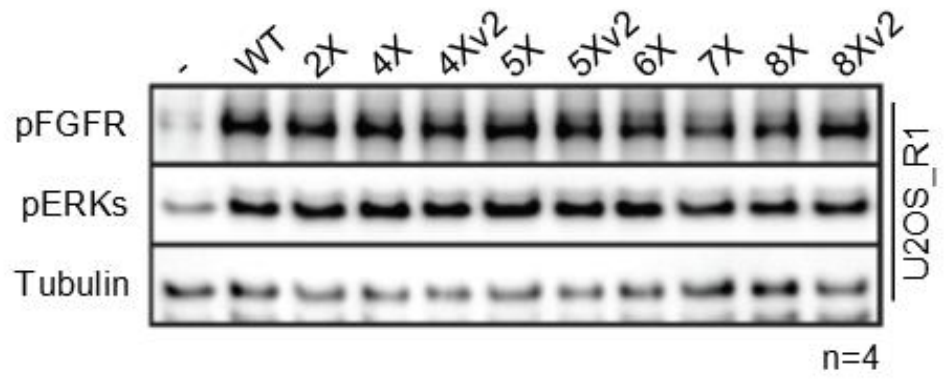

Supplementary Figure 2. Biological activity of multiple FGF2 variants. Activation of downstream signaling by multiple FGF2 variants in U2OS_R1 cells. Serum-starved cells were treated with proteins at a concentration of 10 ng/mL. After 15 min, cell lysates were collected and subjected to SDS-PAGE followed by western blot analysis. Phosphorylation of FGFR and ERKs were used to confirm biological activity of the obtained variants. Tubulin served as a loading control; (n=4).

Supplementary Figure 3

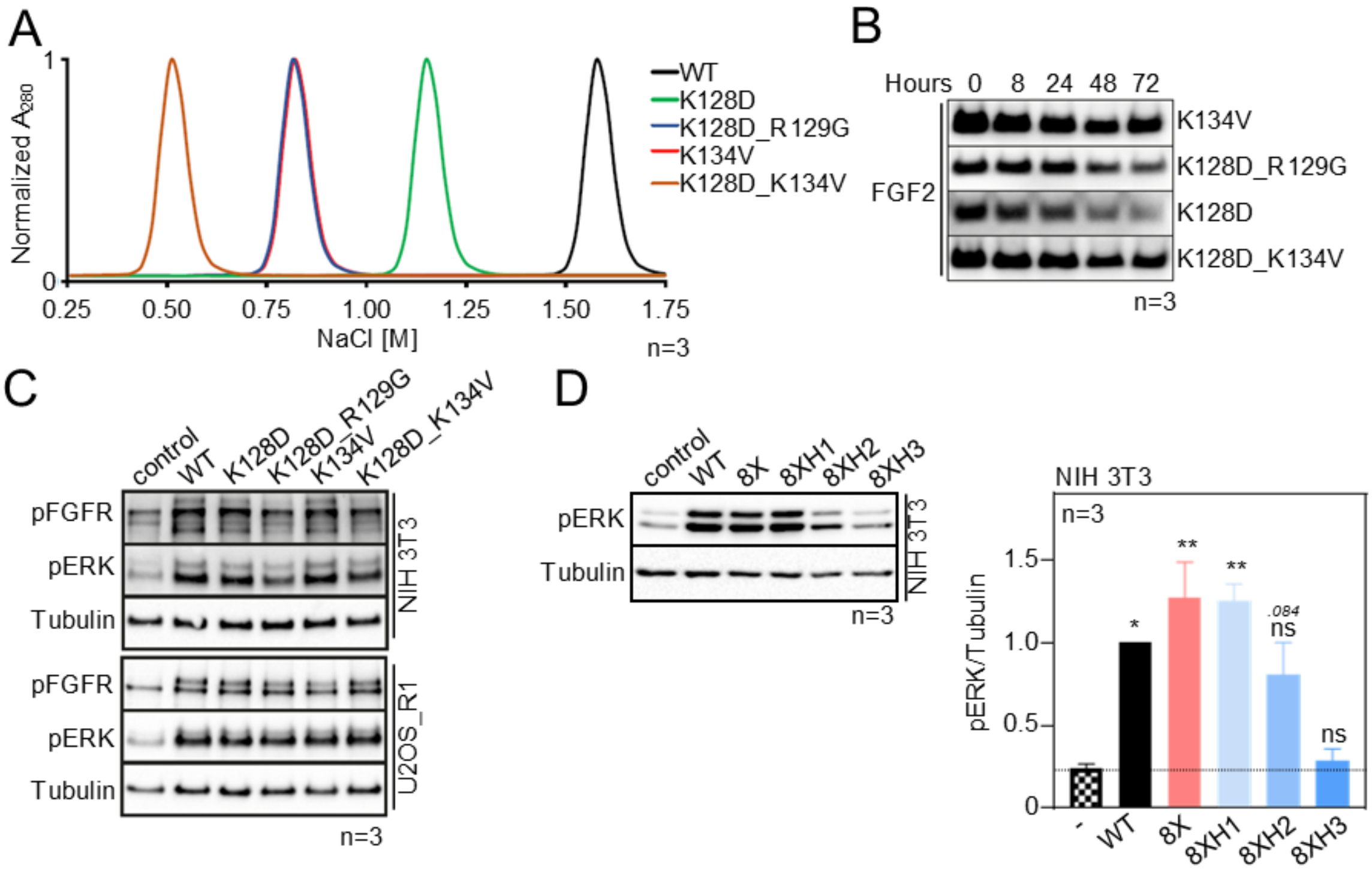

Supplementary Figure 3. Characteristics of FGF2 variants with reduced affinity for heparin. (**A**) Schematic elution profile of FGF2 variants with reduced affinity for heparin. Proteins were loaded onto HiTrap Heparin HP column and eluted in a 0.05-2.05 M NaCl gradient; (n=3). (**B**) Western blot analysis of degradation of FGF2 variants with reduced affinity for heparin. 1 µg of protein was incubated in complete NIH 3T3-conditioned medium over the cells, and at the indicated time points, samples were collected and subjected to SDS-PAGE followed by western blot with anti-FGF2 antibody; (n=3). (**C**) Activation of downstream signaling by FGF2 variants with reduced affinity for heparin. Serum-starved NIH 3T3 and U2OS_R1 cells were treated with protein at a concentration of 10 ng/mL in the presence of 10 U/mL heparin. After 15 min, cell lysates were collected and subjected to SDS-PAGE followed by western blot analysis. Phosphorylation of FGFR and ERKs was used to confirm the biological activity of the obtained variants. Tubulin served as a loading control; (n=3). (**D**) Activation of downstream signaling by stable FGF2 variant (containing 8X mutations) with reduced affinity for heparin. Serum-starved NIH 3T3 cells were treated with the protein at a concentration of 10 ng/mL. After 15 min, cell lysates were collected and subjected to SDS-PAGE followed by western blot analysis. ERK phosphorylation was used to confirm the biological competence of the obtained variants. Tubulin served as a loading control. Statistical analysis was performed using one-way ANOVA, $*p \leq 0.05$, $**p \leq 0.01$; (n=3); data are presented as mean±SEM.

Supplementary Figure 4

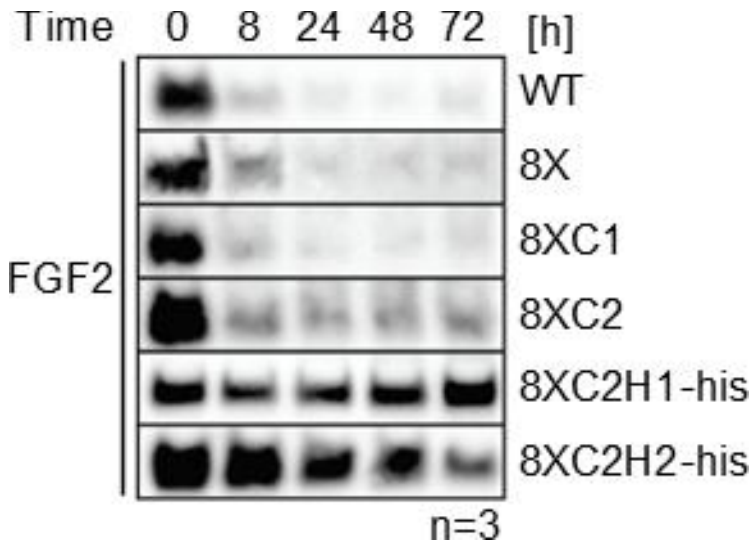

Supplementary Figure 4. Proteolytic stability of selected multiple FGF2 variants. Analysis of degradation of stable FGF2 variants in NIH 3T3-conditioned medium. 1 µg of proteins were incubated in complete NIH 3T3-conditioned medium over the cells and at the indicated time points samples were collected and subjected to SDS-PAGE followed by western blot analysis with anti-FGF2; (n=3).

Supplementary Figure 5

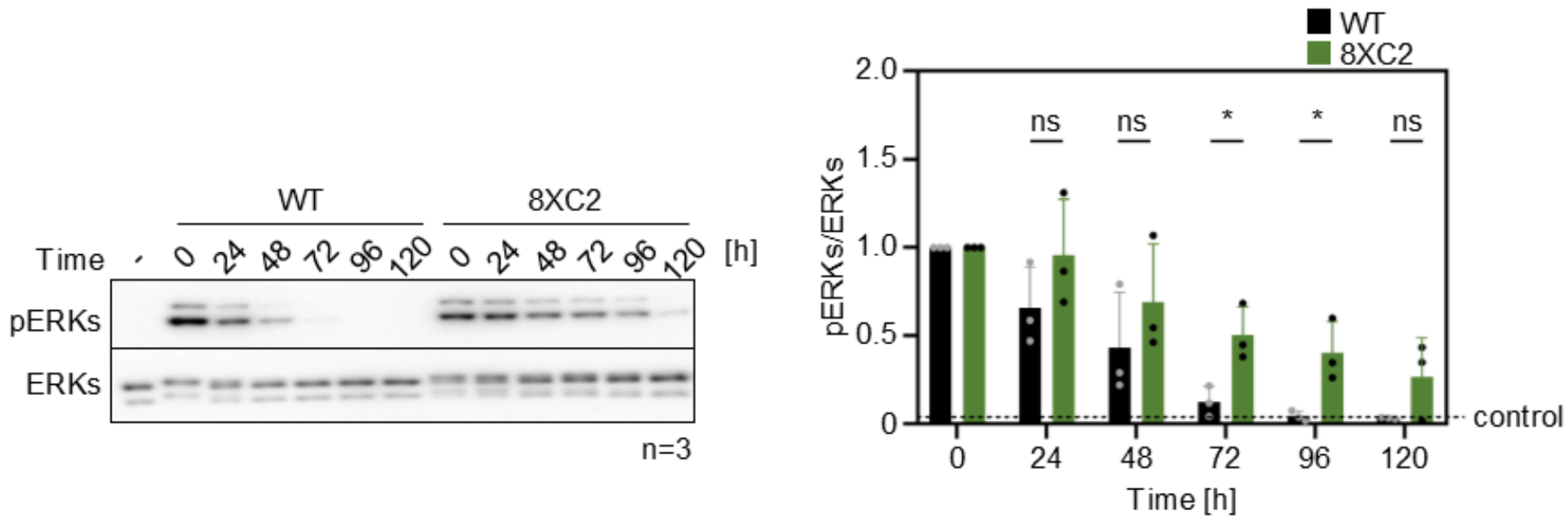

Supplementary Figure 5. Activity in RCS cells of the stable mutant after incubation with 3T3-L1 adipocytes. To study the degradation of FGF2 variants over five days, differentiated 3T3-L1 cells were seeded in 24-well plates and treated with individual proteins at a concentration of 1 µg/mL. RCS cells were then stimulated for 30 min with 10 µl/mL of culture medium of 3T3-L1 cells from each day of incubation with FGF2 variants in the presence of 10 U/mL heparin. Cells were then collected, lysed in Laemmli sample buffer and analyzed. FGF2 variants were pre-incubated with 3T3-L1 adipocytes medium for the indicated time before being added to RCS cells for 30 min. Phosphorylated (p) ERKs were detected by western blot, with total ERKs serving as loading controls. Densitometric analysis of the signal of pERKs and ERKs is shown, values relative to the activity of freshly added wild-type FGF2. Statistical analysis was performed using *t-test*, *$p \leq 0.05$, (n=3); data are presented as mean±SD.

Supplementary Figure 6

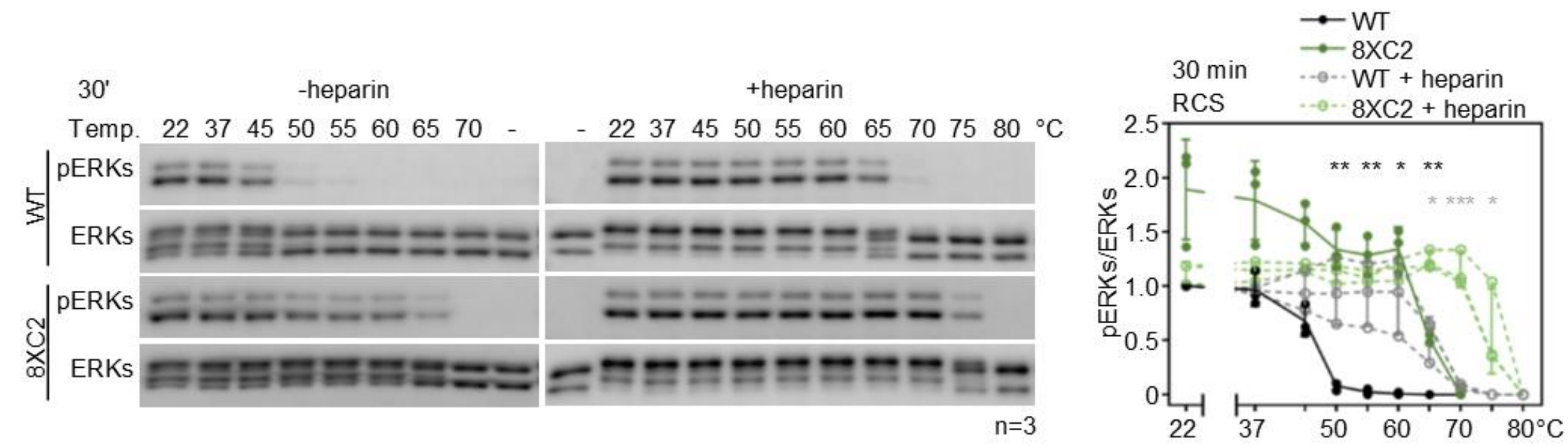

Supplementary Figure 6. Ability to activate signaling pathways by a stable variant of FGF2 incubated at elevated temperatures. RCS cells were treated for 30 min with 20 ng/mL of pre-incubated for 1 h at elevated temperature (37-80°C) FGF2 variants (in the presence or absence of 1 μg/mL heparin). Signaling analysis was performed by detecting phosphorylated (p) ERKs by western blot, with total ERKs served as loading controls. Densitometric analyses versus signaling for FGF2 WT at 22°C. Statistical analysis was performed using *t-test*, *p≤0.05, (n=3); data are presented as mean±SEM.

Supplementary Figure 7

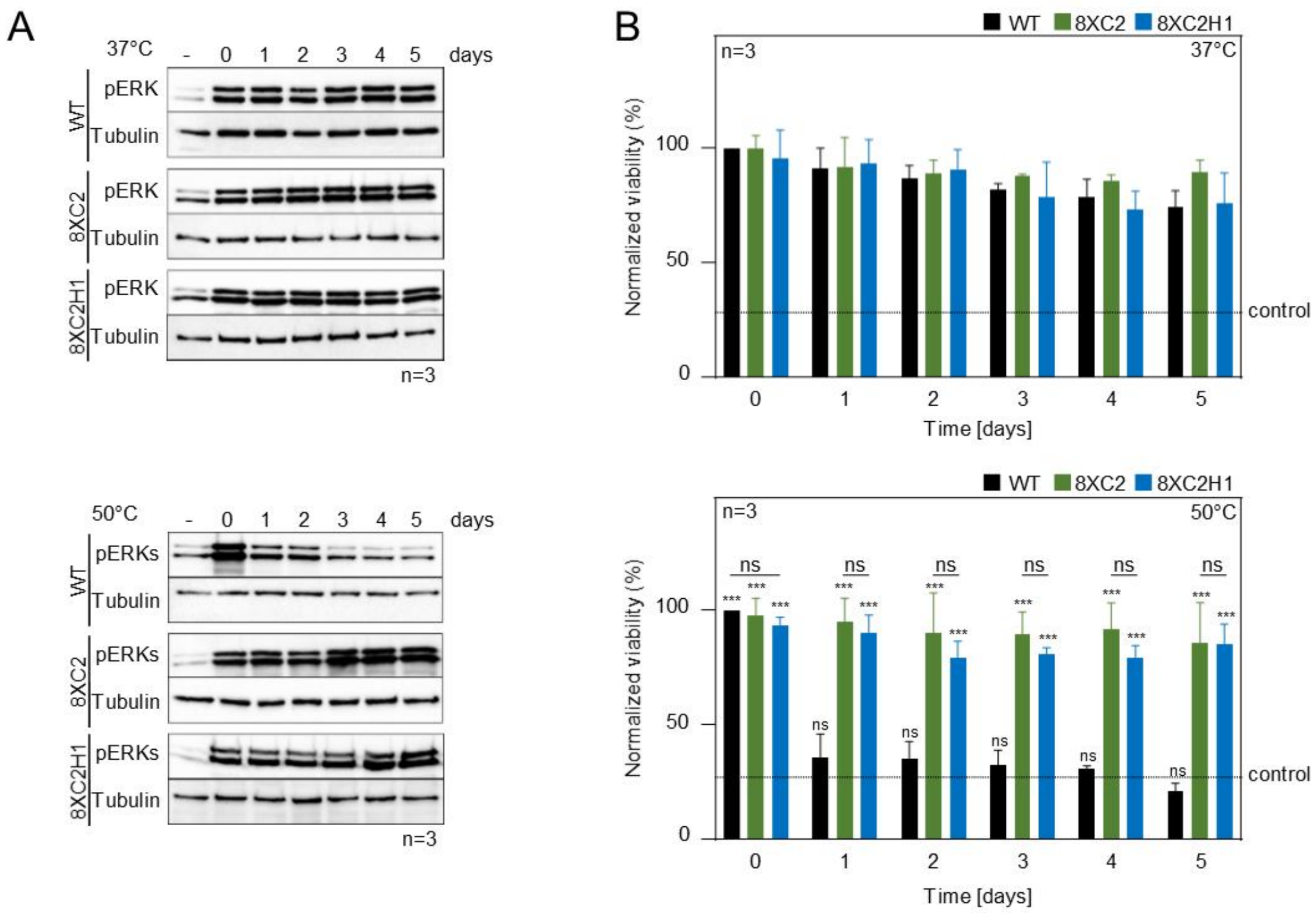

Supplementary Figure 7. The effect of incubation at elevated temperature on the activation of downstream signaling and the mitogenic activity of FGF2 variants. (A) NIH 3T3 cells were exposed for 15 min to a concentration of 10 ng/mL of FGF2 variants that had been pre-incubated for 0-5 days at elevated temperature (37 or 50°C). Signaling analysis was performed by detecting phosphorylated (p) ERKs by western blot, with tubulin serving as a loading control. (B) Viability of NIH 3T3 cells following stimulation with fresh stable FGF2 mutants and proteins preincubated at 37°C or 50°C for 5 days. Serum-starved cells were treated with FGF2 mutants at concentration 10 ng/mL in the presence of 10 U/mL of heparin. After 48 h, cell viability was assessed by AlamarBlue assay. The maximum response for fresh wild-type FGF2 (day 0) was set as 100%. Data are presented as mean±SD; two-way ANOVA, ns>0.05, ***p≤0.001 (n=3).

Supplementary Figure 8

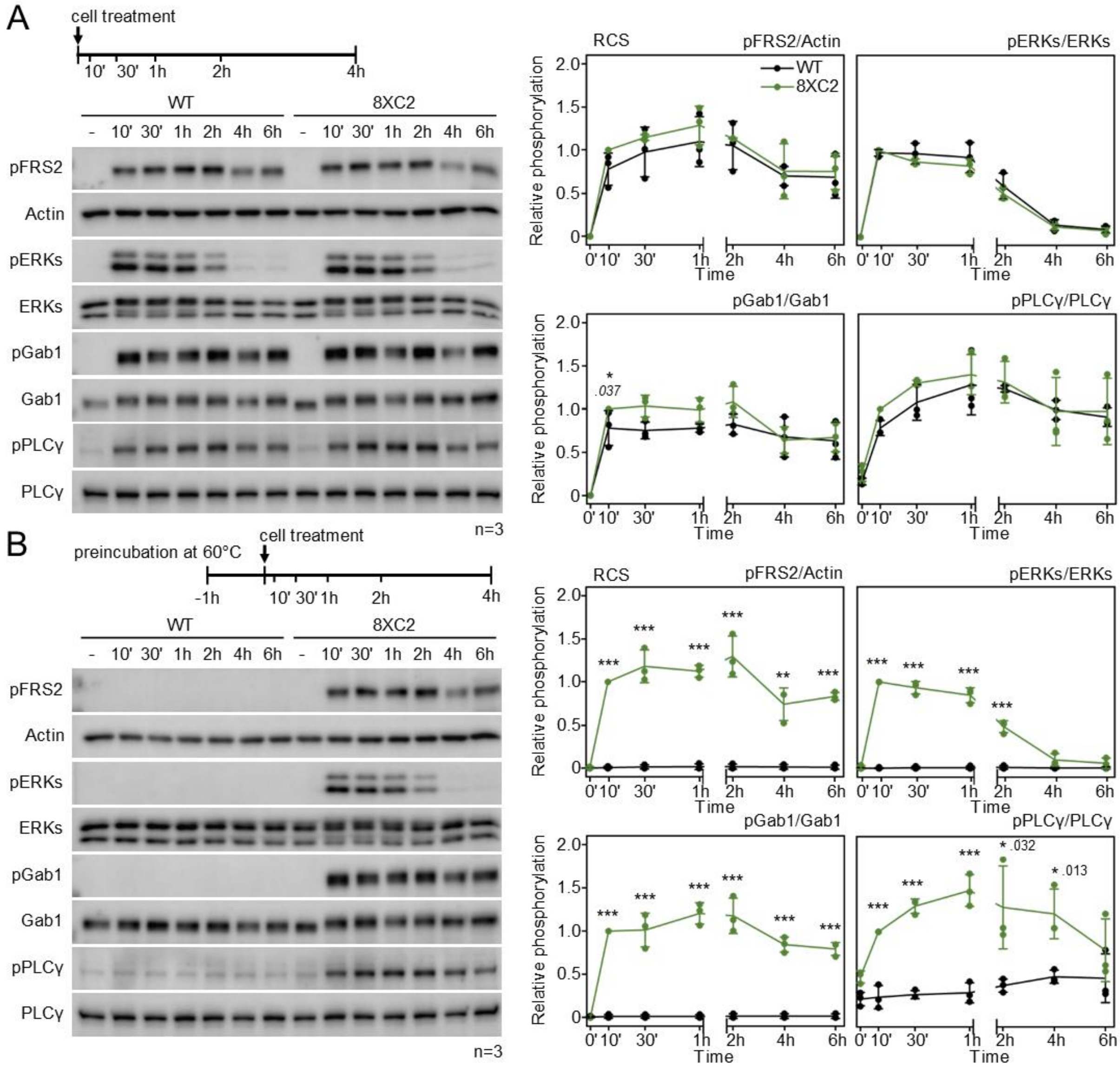

Supplementary Figure 8. The enhanced thermal stability of modified FGF2 preserves its signaling activity in RCS cells. (A, B) RCS cells were treated with (A) fresh FGF2 or (B) FGF2 pre-incubated at 60°C/1 h. Phosphorylated (p) ERKs, FRS2, Gab1 and PLCγ were detected by western blot; total ERKs, PLCγ, Gab1 and actin served as loading controls. Densitometric analyses of p-protein signal are shown, data normalized to a 10 min FGF2 8XC2 sample. Data are presented as mean±SD; *t*-test, *p≤0.05, ***p≤0.001; n, number of independent experiments.

Supplementary Figure 9

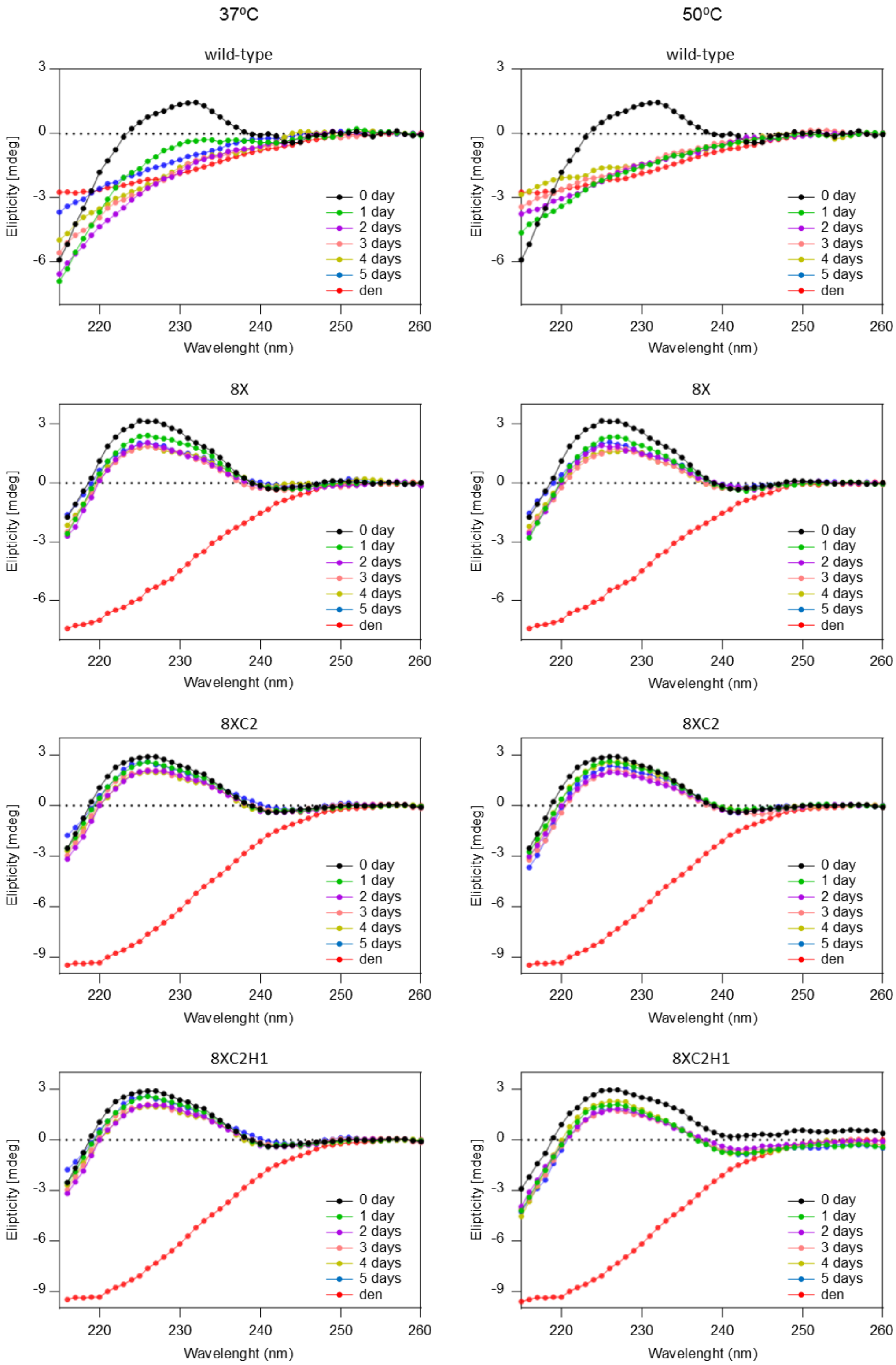

Supplementary Figure 9. Temperature sensitivity of FGF2 variants assessed by circular dichroism during a 5-day incubation at 37°C and 50°C. Circular dichroism (CD) spectra were recorded in the wavelength range of 200–260 nm at a protein concentration of 2 μM. Measurements were performed daily over a 5-day period. Additionally, thermal denaturation (den) was performed as a negative control. Representative spectra are shown (n=3).

Supplementary Figure 10

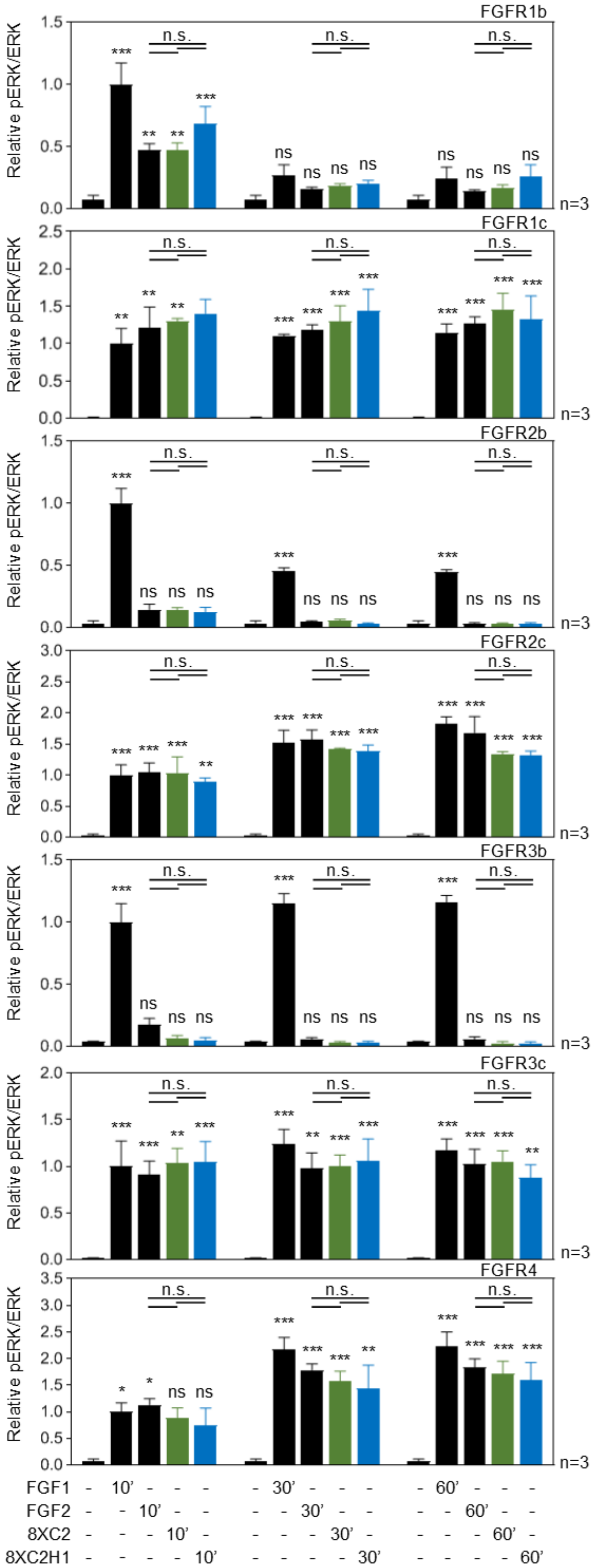

Supplementary Figure 10. Quantitative analysis of the specificity of stable FGF2 variants in RCS cells based on the results shown in Figure 2B. Quantitative data from Fig. 2B are presented as plots based on three independent experiments (n=3). Data were normalized to the maximum FGF1 response and shown as mean±SEM. Statistical analysis was performed using two-way ANOVA multiple comparisons test (ns, $p > 0.05$; *$p \leq 0.05$, **$p \leq 0.01$, ***$p \leq 0.001$).

Supplementary Figure 11

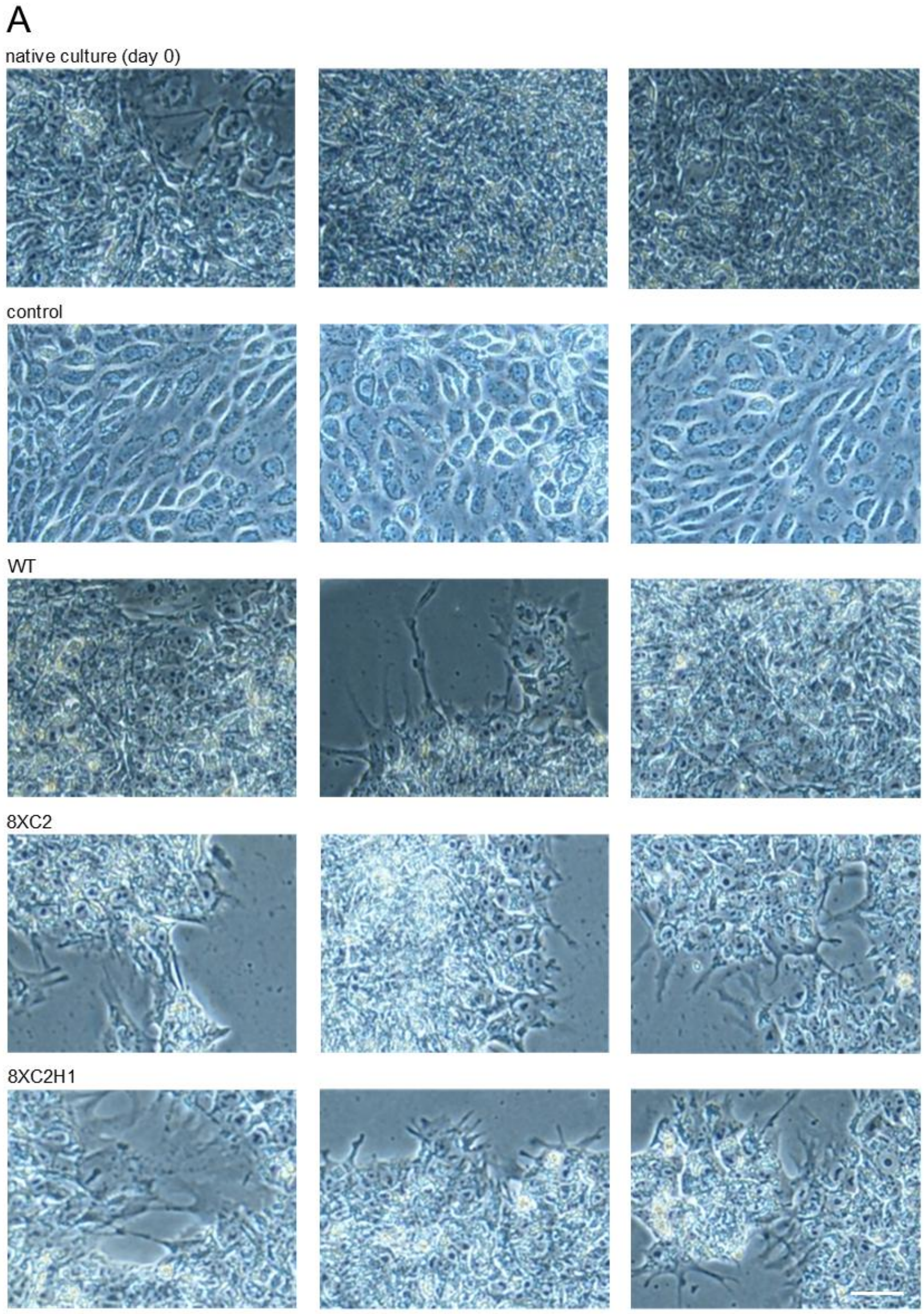

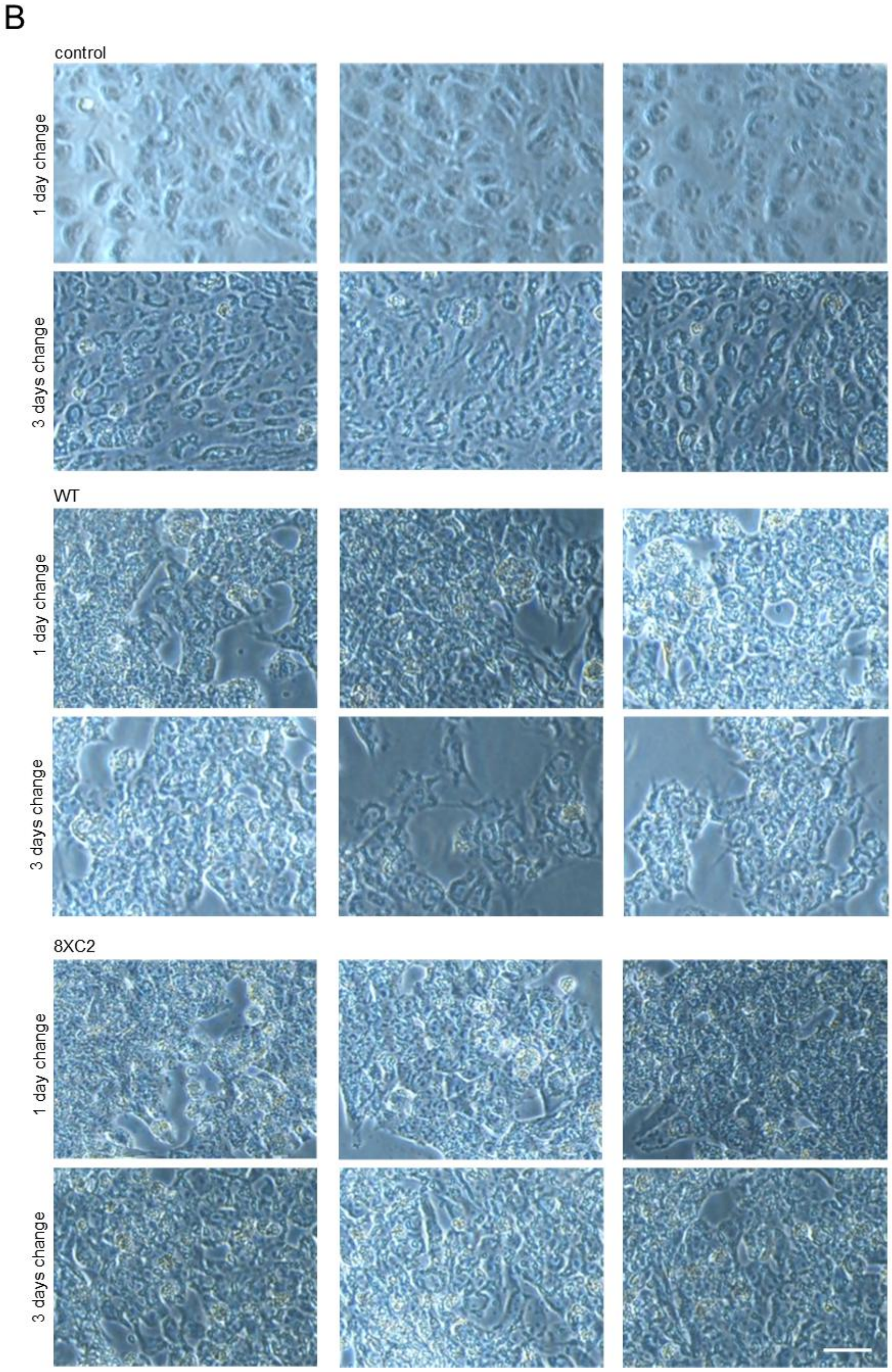

Supplementary Figure 11. Increased stability of FGF2 prolongs maintenance of iPS cell pluripotency. (A) Morphology of iPS cells cultured on Matrigel in conditioned medium without FGF (control) or supplemented with FGF2 variants (wild-type, 8XC2, 8XC2H1) over three passages (10-12 days). An additional image of cells at day 0 is also shown. The medium was changed daily, scale bar 50 µm. (**B**) Morphology of iPS cells on Matrigel in conditioned medium without FGF (control) or supplemented with FGF2 variants (wild-type, 8XC2) over five passages (30-36 days) with medium change daily or every three days. Scale bar 50 µm.

Supplementary Figure 12

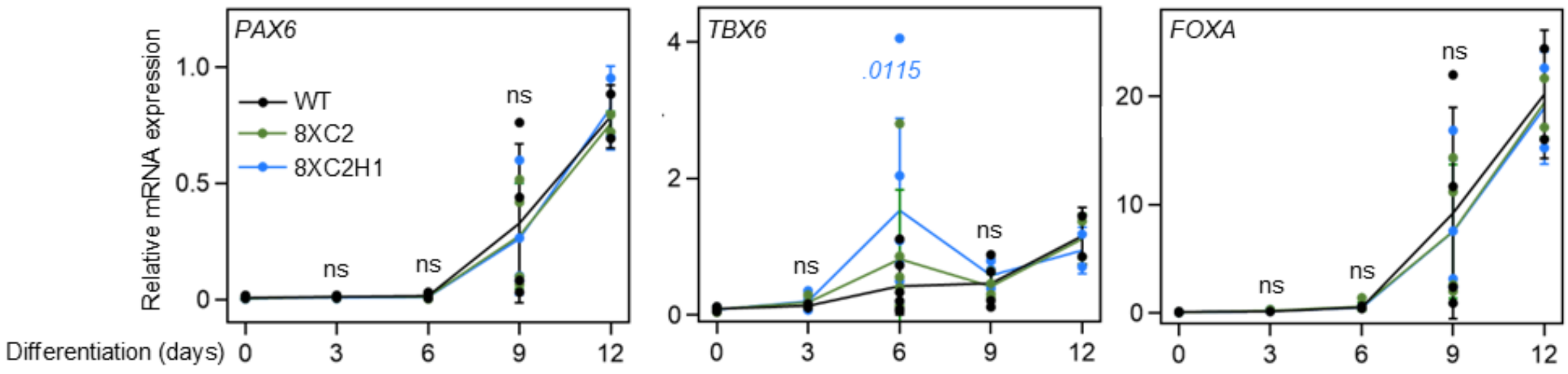

Supplementary Figure 12. FGF2 stable variants do not interfere with the differentiation of human iPS cells. hiPS (cl.4) cells were cultured in the presence of 100 ng/mL of FGF2 for 9 days, after which FGF2 was removed and cells were further grown until day 12. Spontaneous differentiation during the culture period in FGF2-free media was monitored by qPCR for ectoderm (*PAX6*), mesoderm (*TBX6*) and endoderm (*FOXA*) markers. Data are presented as mean±SD; one-way ANOVA; ns, non-significant. N, number of biological replicates (n=3 for days 0-9, n=2 for day 12).

Supplementary Table 1. List of antibodies used in the study

Protein	*Provider*	*Cat. No.*	*Method*
Actin	Cell Signaling	3700	WB
Caveolin 1	Cell Signaling	3238	WB
Collagen 2	Abcam	AB34712	WB
pERK	Cell Signaling	9101	WB
$pERK^{T202/Y204}$	Cell Signaling	4376	WB
ERK	Cell Signaling	9102	WB
$pFGFR\text{-}1^{Tyr653/Tyr654}$	Millipore	06-1433	WB
pFRS2	Cell Signaling	3864	WB
$pGAB1^{Y627}$	Cell Signaling	3231	WB
GAB1	Cell Signaling	3232	WB
Lamin AC	Cell Signaling	2032	WB
$pPLC\gamma^{Tyr783}$	Cell Signaling	2821	WB
PLCγ	Cell Signaling	2822	WB
γTubulin	Sigma	T6557	WB
FGF2	Santa Cruz	sc-74412	WB
mouse IgG	Sigma Aldrich	A6782	2nd, WB
rabbit IgG	Sigma Aldrich	A6667	2nd, WB
mouse IgG	Jackson ImmunoResearch	115-035-003	2nd, WB
rabbit IgG	Jackson ImmunoResearch	111-035-144	2nd, WB

WB, western blot; 2nd, secondary antibody.

Supplementary Table 2. List of oligonucleotides used in the study for quantitative real-time PCR

Target	*type*	*Sequence 5‘-3‘*
GAPDH	forward	GAAATCCCATCACCATCTTCC
	reverse	GGCTGTTGTCATACTTCTCAT
POU5F1	forward	GCAAAGCAGAAACCCTCGT
	reverse	ACACTCGGACCACATCCTTC
NANOG	forward	CCTATGCCTGTGATTTGTGG
	reverse	CTGGGACCTTGTCTTCCTTT
PAX6	forward	GTCCATCTTTGCTTGGGAAA
	reverse	TAGCCAGGTTGCGAAGAACT
TBX6	forward	TCATCTCCGTGACAGCCTACCA
	reverse	CCGCAGTTTCCTCTTCACACGG
FOXA2	forward	GGAACACCACTACGCCTTCAAC
	reverse	AGTGCATCACCTGTTCGTAGGC

Supplementary Table 3. Comparison of denaturation parameters of $FGF2_{short}$ and $FGF2_{long}$ determined by circular dichroism

FGF2 variant	$FGF2_{short}$			$FGF2_{long}$		
	ΔT_m (°C)	T_m (°C)	ΔH_{den} (kcal/mol)	ΔT_m (°C)	T_m (°C)	ΔH_{den} (kcal/mol)
wild-type		56.7±0.8	98.8±4.2		56.2±0.7	102.4±8.0
K61I	4.7	61.4±0.8	101.3±18.4	4.8	61.0±0.3	116.3±3.6
N111G	4.9	61.6±0.04	114.0±3.9	4.2	60.4±0.7	106.4±8.1
2X (K61I/N111G)	9.1	65.8±0.2	116.4±5.1	7.9	64.1±0.03	88.9±6.7
8X (R31L/E54D/H59Y/K61I/E67V/N111G/T121P/S137P)	22.6	79.3±0.8	114.4±5.7	23.0	79.2±0.5	132.9±15.2

ΔT_m - difference in melting temperature between mutant and wild-type; T_m - melting temperature; ΔH_{den} - enthalpy of denaturation, mean±SD; (n=3).

Supplementary Table 4. Denaturation parameters of $FGF2_{short}$ variants with enhanced stability determined by circular dichroism

$FGF2_{short}$	ΔT_m (°C)	T_m (°C)	ΔH_{den} (kcal/mol)
wild-type		56.7±0.8	98.8±4.2
K86D	0.2	56.9±0.1	97.45±2.3
A66S	0.4	57.1±0.5	103.4±7.9
S56N	0.5	57.2±0.3	97.0±6.1
F39Y	0.6	57.3±0.4	103.1±2.0
K30T	0.7	57.4±0.3	105.6±0.5
H44R	0.7	57.4±0.1	93.2±1.1
A66P	0.7	57.4±0.2	95.0±9.9
K95P	0.8	57.5±0.8	94.1±5.8
Q63E	0.8	57.5±0.2	76.6±16.7
K95E	0.8	57.5± 0.8	102.6±17.2
S122N	1.2	57.9±0 .1	108.0±2.6
T130D	1.2	57.9±0.2	101.6±1.6
S137P	2.2	58.9±0.2	118.8±13.6
Q63I[&]	2.6	59.3±0.3	97.6±6.5
E67V	3.0	59.7±0.1	105.9±8.2
E54D	3.2	59.9±0.1	111.1±4.7
H59Y	4.2	60.9±0.04	111.2±0.9
I60T	4.3	61.0±0.6	105.2±2.5
K61I	4.7	61.4±0.8	101.3±18.4
N111G	4.9	61.6±0.04	114.0±3.9

ΔT_m - difference in melting temperature between mutant and wild-type; T_m - melting temperature; ΔH_{den} - enthalpy of denaturation; mean±SD; (n=3, except [&] where mean ±1/2 range, n=2); blue - mutations used in multiple variants.

Supplementary Table 5. Denaturation parameters of $FGF2_{short}$ variants with reduced stability determined by circular dichroism

$FGF2_{short}$	ΔT_m (°C)	T_m (°C)	ΔH_{den} (kcal/mol)
wild-type		56.7	98.8
V71E	-9.3	47.4	82.8
F40H	-6.9	49.8	39.4
R81Y	-6.9	49.8	63.9
F104R	-6.7	50.0	86.2
H44D	-5.2	51.5	91.7
S137N	-1.8	54.9	97.0
A79S	-1.6	55.1	94.0
R118E	-1.5	55.2	96.9
S122D	-1.3	55.4	96.5
S109P	-1.2	55.5	85.7
G140R	-1.0	55.7	84.9
S73R	-0.9	55.8	85.6
V97F	-0.7	56.0	96.8
L147H	-0.6	56.1	94.6
N80G	-0.4	56.3	112.2
S122G	-0.3	56.4	82.3

ΔT_m - difference in melting temperature between mutant and wild-type; T_m - melting temperature; ΔH_{den} - enthalpy of denaturation; (n≥1).

Supplementary Table 6. **Denaturation parameters of multiple variants of $FGF2_{short}$ with enhanced stability determined by circular dichroism**

$FGF2_{short}$	ΔT_m (°C)	T_m(°C)	ΔH_{den} (kcal/mol)
wild-type		56.7±0.8	98.8±4.2
2X (K61I/N111G)	9.1	65.8±0.2	116.4±5.1
4X (E54D/H59Y/K61I/N111G)	14.9	71.6±0.3	118.2±12.4
4Xv2 (H59Y/K61I/E67V/N111G)	13.5	70.2±1.5	124.8±4.9
5X (E54D/H59Y/K61I/E67V/N111G)	15.0	71.7±2.9	118.0±20.1
5Xv2 (H59Y/K61I/E67V/N111G/S137P)	15.8	72.5±0.2	108.3±0.1
6X (H59Y/K61I/Q63I/E67V/N111G/S137P)	14.2	70.9±0.3	98.8±11.6
7X (R31L/E54D/H59Y/K61I/E67V/N111G/T121P)	20.3	77.0±1.4	103.2±24.4
8X (R31L/E54D/H59Y/K61I/E67V/N111G/T121P/S137P)	22.6	79.3±0.8	114.4±5.7
8Xv2 (R31L/E54D/H59Y/I60T/K61I/E67V/N111G/S137P)	19.4	76.1±0.3	123.7±6.9

ΔT_m - difference in melting temperature between mutant and wild-type; T_m - melting temperature; ΔH_{den} - enthalpy of denaturation; mean±SD; (n=3).

Supplementary Table 7. Characterization of $FGF2_{short}$ variants with reduced heparin binding

$FGF2_{short}$	ΔT_m (°C)	T_m(°C)	ΔH_{den} (kcal/mol)	NaCl (M)
wild-type		56.7±0.8	98.8±4.2	1.5
K134V	-3.8	52.9±0.8	90.1±6.9	0.88
K128D_R129G	2.7	59.4±0.01	94.3±3.2	0.88
K128D	3.2	59.9±1.2	106.5±5.3	1.23
K128D_K134V	5.2	61.9±0.4	94.2±3.0	0.6

Denaturation parameters determined by circular dichroism measurements and affinity for heparin expressed as the concentration of NaCl required for elution from the heparin column; (SD<0.1; n=3). ΔT_m - difference in melting temperature between mutant and wild-type; T_m - melting temperature; ΔH_{den} - enthalpy of denaturation, blue - mutation used in multiple variant; (n=3).

Supplementary Table 8. Characterization of multiple stable $FGF2_{short}$ variants with reduced heparin binding

$FGF2_{short}$	ΔT_m (°C)	T_m (°C)	ΔH_{den} (kcal/mol)	NaCl (M)	K_D (M)
wild-type		56.7±0.8	98.8±4.2	1.5	6.35E-08
8X	22.6	79.3±0.8	114.4±5.7	1.59	1.79E-07
wild-type-his		52.6±2.0	95.2±6.6	1.5	9.21E-08
8X_K128D-his	28.8	81.4±1.8	90.4±14.0	0.94	5.48E-08
8X_K128D_K134V-his	29.9	82.5±2.4	110.9±7.5	0.42	6.99E-08
8X_K128D_K134V_R129G-his	30.1	82.7±2.2	90.8±15.2	0.3	5.00E-08

Denaturation parameters determined by circular dichroism measurements; (n≥2) and affinity for heparin expressed as the concentration of NaCl required for elution from the heparin column; (n=3). ΔT_m - difference in melting temperature between mutant and wild-type; T_m - melting temperature; ΔH_{den} - enthalpy of denaturation (mean±SD; n=3), blue - mutations that disturb heparin affinity. K_D - equilibrium dissociation constant, calculated from fitted saturation binding curves to the FGFR1c receptor; (n=3).

Supplementary Table 9. Denaturation parameters of stable multiple variants of $FGF2_{long}$ determined by circular dichroism

$FGF2_{long}$	Mutations	ΔT_m (°C)	T_m (°C)	ΔH_{den} (kcal/mol)
wild-type			56.2±0.7	102.4±8.0
8X	R31L/E54D/H59Y/K61I/E67V/N111G/T121P/S137P	23.0	79.2±0.5	132.9±15.2
8XC1	R31L/E54D/H59Y/K61I/E67V/C96S/N111G/T121P/S137P	22.9	79.1±0.1	144.9±10.1
8XC2	R31L/E54D/H59Y/K61I/E67V/C78S/C96S/N111G/T121P/S137P	22.1	78.3±0.1	139.7±3.3
wild-type-his			53.9±0.1	94.9±2.0
8XH1-his	R31L/E54D/H59Y/K61I/E67V/N111G/T121P/K128D/S137P	29.5	83.4±0.1	151.1±3.4
8XH2-his	R31L/E54D/H59Y/K61I/E67V/N111G/T121P/K128D/K134V/S137P	23.8	77.7±0.8	82.7±4.9
8XC1H1-his	R31L/E54D/H59Y/K61I/E67V/C96S/N111G/T121P/K128D/S137P	28.0	81.9±0.6	120.1±10.2
8XC1H2-his	R31L/E54D/H59Y/K61I/E67V/C96S/N111G/T121P/K128D/K134V/S137P	30.6	84.5±0.7	115.1±9.7
8XC2H1-his	R31L/E54D/H59Y/K61I/E67V/C78S/C96S/N111G/T121P/K128D/S137P	27.8	81.7±1.0	135.8±6.2
8XC2H2-his	R31L/E54D/H59Y/K61I/E67V/C78S/C96S/N111G/T121P/K128D/K134V/S137P	27.9	81.8±1.4	82.9±57.1

ΔT_m - difference in melting temperature between mutant and wild-type; T_m - melting temperature; ΔH_{den} - enthalpy of denaturation; mean±SD; (n=3); blue - mutations that reduce heparin affinity, red - mutations that improve thiol chemistry.

Supplementary Table 10. Comparison of denaturation parameters of stable multiple variants of FGF2, 8XC2H1 and G3 (Dvorak *et al.*, 2018), determined using circular dichroism

FGF2 variant	Mutations	ΔT_m (°C)	T_m (°C)	ΔH_{den} (kcal/mol)
wild-type$_{short}$			56.7±0.8	98.8±4.2
G3	R31L/V52T/E54D/H59F/L92Y/S94I/C96N/S109E/T121P	19.9	76.6±0.3	118.5±4.1
wild-type$_{long}$-his			53.9±0.1	94.9±2.0
8XC2H1$_{long}$-his	R31L/E54D/H59Y/K61I/E67V/C78S/C96S/N111G/T121P/K128D/S137P	27.8	81.7±1.0	135.8±6.2

ΔT_m - difference in melting temperature between mutant and wild-type; T_m - melting temperature; ΔH_{den} - enthalpy of denaturation; mean±SD; (n=3); G3 - short version of the stable FGF2 variant constructed by Dvorak *et al.* (2018).

Supplementary Table 11. The effect of heparin on the thermal stability of FGF2 variants

FGF2 variant	- heparin		+ heparin		
	T_m (°C)	ΔH_{den} (kcal/mol)	T_m (°C)	ΔH_{den} (kcal/mol)	ΔT_m ((+heparin)-(-heparin)) (°C)
wild-type	56.7±0.8	98.8±4.2	85.2±0.4	102.8±2.6	28.5
8X	79.2±0.5	132.9±15.2	99.5±0.9	98.9±18.7	20.3
8XC2	78.3±0.1	139.7±3.3	97.0±0.6	126.4±0.6	18.7
8XC2H1	81.7±1.0	135.8±6.2	87.6±0.7	128.2±12.1	5.9

T_m - melting temperature; ΔH_{den} - enthalpy of denaturation; ΔT_m ((+heparin)-(-heparin)) - difference in denaturation temperatures in the presence and absence of heparin; mean±SD; (n=3); heparin was used at a concentration of 1 µM (assuming an average molecular weight of 15 kDa), which represented a twofold molar excess relative the protein concentration (0.5 µM).

Supplementary Table 12. The effect of heparin on the affinity of stable FGF2 variants for FGFR1c

**FGF2 variant**	**K_D±SD**	
	**- heparin**	**+ heparin**
WT	15.3±2.5	14.3±3.3
8XC2	15.3±3.5	9.3±0.3
8XC2H1	48.0±19.7	13.3±1.7

BLI analysis of the affinity of FGF2 variants for FGFR1-Fc (IIIc isoform) in the presence and absence of 1 µM heparin. FGFR1-Fc was immobilized on a ProA sensor, and its interactions (association and dissociation) with selected FGF2 mutants were analyzed over a concentration range of 4-512 nM. Equilibrium dissociation constants (K_D) were calculated from saturation binding curves; mean±SD; (n=3).
